# Green synthesis of nanosized *N,N*'-bis(1-naphthylidene)-4,4'-diaminodiphenylmethane and its metal (II) complexes and evaluation of their biological activity

**DOI:** 10.1038/s41598-022-25650-z

**Published:** 2022-12-07

**Authors:** Hammed H. A. M. Hassan, Hend M. Hussein, Amel F. Elhusseiny

**Affiliations:** 1 Department of Chemistry, Faculty of Science, Alexandria University, Moharram Beck, P.O. Box 2, Alexandria, 21568 Egypt; 2 Pharmacology and Therapeutics Department, Faculty of Pharmacy, Pharos University, Canal El Mahmoudia Street, Alexandria, 21311 Egypt

**Keywords:** Microbiology, Chemistry

## Abstract

Condensation of ecofriendly synthesized 4,4’-methanedianiline with 2-hydroxy-1-naphthaldehyde produced a (1:1) octopus-like Schiff base mixed ligand. Reaction with Co(OAc)_2_⋅H_2_O, NiCl_2_⋅6H_2_O, Cu(OAc)_2_⋅H_2_O and Zn(OAc)_2_⋅2H_2_O metals furnished their complexes in high yield and purity. All new structures were fully characterized by various spectroscopic and spectrometric measurements. The complexes exhibited high thermal stability up to 700 °C, leaving nearly 40% of their mass as residues. Antimicrobial screening results exhibited moderate activities towards all studied microbes. Antioxidant screening was concentration dependent, and their activities were in the order Ni(II) > Zn(II) > Cu(II) > Co(II) complexes. The NO inhibitory effect revealed that the nickel complex exhibited the highest activity, whereas the cobalt complex showed the lowest inhibition. All compounds showed a significant lipid peroxidation inhibitory effect against oxidative stress. The complexes significantly diminished the TBARS level, and the nickel complex exhibited the highest inhibition at p < 0.01. Antioxidants stress the oxidative damage induced by iron, indicating that the nickel complex has the highest reducing activity. The inhibitory effect against acetylcholine esterase showed that the copper complex has the highest activity. Membrane stabilization activities clearly indicated that most compounds can improve the integrity of the cells and stability of their membrane, and this result may be related to their antioxidant capacity to protect against cytotoxicity. The nickel complex exhibited a stronger total antioxidant capacity than the other complexes. The biological and antioxidant capacities of these complexes may make them promising candidates in pharmaceutical applications.

## Introduction

Clay minerals are an efficient new green technology in a variety of industrial processes because of their different set of properties, such as being inexpensive, environmentally friendly, nontoxic, reusable, and mid-catalyst. They are also very effective catalysts for a wide variety of regio- and selective organic reactions^1^. Kaolinite is a 1:1 layer phyllosilicate clay mineral, and its chemical formula is Si_2_Al_2_O_5_ (OH)_4_, Al_2_O_5_Si_2_.2H_2_O or Al_2_O_3_.2Si_4_.2H_2_O^1^ along with other minor metal oxide impurities^2^. Because of its acidic properties, natural kaolinite has been used in many chemical and industrial reactions^1^. 4,4’-Methylenedianiline has been extensively used in many applications, including curing agents and chain extenders in polymers. In this work, we highlight green heterogeneous catalysts for methylenedianiline synthesis and report, for the first time, plausibly the reaction mechanism. The insertion of a methylene group, as in the case of methylenedianiline, causes the dilution of aromatic nuclei, a source of polar forces, and improves processability and properties, such as solubility and melting of the product containing such a moiety^3^.

Transition metal ions are involved in many biological processes crucial for sustaining life. These metals can serve as cofactors in proteins, enabling their biological function, regulating their activity, and/or stabilizing their structure^4–6^. Copper is an essential element for life because it is associated with several copper-dependent enzymes that are key in biological processes in mammals. Elevated copper levels in plasma can be important for the etiology of some illness. For example, copper ions are closely involved in neurodegenerative disorders, especially in Parkinson's disease. Copper (II) complexes possessed various activities such as antiulcer, antiamoebic, antidiabetic, anticonvulsant, anti-inflammatory, antimicrobial and antitumor^7^. Cobalt like copper is an essential trace element for higher organisms. It is required in the active center of coenzymes, the so-called cobalamins, especially Vitamin B12 which regulates indirectly the synthesis of DNA. Moreover, there are at least eight cobalt-dependent proteins. Cobalamins alone are pharmaceutical agents and are treated in pathologies arising from a lack of vitamin B12. Compared to copper complexes, the cobalt complexes are limited in medical usage. Such complexes found activity against leukemia and lymphoma cell lines and bacteria strains. Further biological activity of cobalt complexes involves insulin-like properties, antifungal and antioxidant activity^7^. Zinc is an essential trace element which is found throughout the human body in a variety of tissues, such as skin, bone, liver, muscle, or brain. This element is the most abundant transition metal in the brain after iron, where its concentrations reach 0.1–0.5 mM. Is it essential for the folding of DNA-binding domains of transcription factors and has a variety of effects on the nervous system. It plays a crucial role in regulating the aspects of cellular metabolism, including protein, hormone, transcription, and replication functions. However, overabundant levels of zinc can lead to apoptosis and neuronal death, therefore It is important to regulate the zinc balance to maintain the homeostasis. Elevated extracellular levels of zinc lead to the breakdown of the zinc transporting system of the plasma membrane. Zinc complexes exhibited interesting biological activity such as antimicrobial, anti-inflammatory agents, successfully tested for healing zinc-deficient, chronic, and surgical wounds by local administration^7^. In contrast to other transition metals, only trace concentrations of manganese are found in human serum (< 10 nM) and tissue (< 4 μM). Only a handful of strictly manganese-dependent enzymes are known in both eukaryotes and prokaryotes because manganese in metalloenzymes appears to be readily interchangeable with other divalent cations^1^. Manganese is the oxygen-evolving complex of photosynthetic plans. Its large amounts (0.5 mg/L) and apparently with far greater effectiveness through inhalation, it can cause a poisoning syndrome in mammals with neurological damage which is sometimes irreversible^8^.

In continuation of our interest in the chemistry of biologically active Schiff bases and their metal complexes^9–14^, we investigated a 2:1 Schiff base green synthesis derived from the reaction of 2-hydroxy-1-naphthaldehyde and the readily available 4,4’-methylenedianiline and its metal (II) complexes with four transition metal salts, namely, zinc-, cobalt- and copper acetates and nickel chloride. The diamine used was synthetic using a modified eco-friendly kaolinite clay catalyst as an acid source in aqueous medium with ultrasonic irradiation^15^. Bearing in mind the pronounced biological activity of Schiff base metal complexes, we examined the biological properties of the titled targeted ligand and its metal(II) complexes against the growth of bacteria and pathogenic fungi. The potential antioxidant ability has been evaluated with respect to 1,1-diphenyl-2-picrylhydrazyl (DPPH), reducing power and nitric oxide scavenging ability as well as acetylcholine esterase (AChE) inhibition. Antihemolytic membrane stabilization activities were also tested.

## Experimental

### General

#### Materials

2-Hydroxy-1-naphthaldehyde, aniline (Aldrich), formaldehyde (35–38%; Sigma), 4,4’-diaminodiphenylmethane (Aldrich), ethanol, 1,4-dioxane, dimethyl sulfoxide (DMSO) and diethyl ether were purchased from Aldrich. The inorganic acetate salts of zinc (II), copper (II), cobalt (II) and nickel (II) chloride were obtained from BDH. All the reagents used were of reagent grade quality and used as received without further purification. The natural kaolinitic clay was obtained from the Department of Geology, Faculty of Science, Alexandria University. Distilled water was used in the whole experiments.

#### Methods

Melting points were measured on a Stuart SMP10 digital melting point apparatus and are uncorrected. Elemental analyses were performed at the Micro Analytical Unit, Cairo University. Metal analysis was determined by the atomic absorption technique at the Faculty of Science, Alexandria University. The molar conductivity measurements were carried out using an HI 8033 HANNA conduct meter at 25 °C for a 10^–3^ M solution in DMSO. Magnetic susceptibility measurements were carried out at room temperature in powder form on a Magway Sherwood product Model MK1 magnetic susceptibility balance using a sealed off sample of MnCl_2_ solution as a calibrant. Absorption spectra were measured with a UV 500 UV‒vis spectrometer at room temperature. Infrared spectra (IR, KBr pellets; 3 mm thickness) were recorded on a Perkin-Elmer Infrared Spectrophotometer (FTIR 1650). All spectra were recorded within the wavenumber range of 500–4000 cm^−1^ at 25 °C. The ^1^H-NMR spectra were recorded on a JEOL 500 spectrometer in DMSO-d_6_ solution with TMS as an internal standard. Electron impact mass spectrometry (EI-MS) measurements were determined using a Finnigan SSQ 7000 spectrometer attached to a digital DEC 300 workstation at the central scientific services unit, National Research Center, Dokki, and Cairo, Egypt. The X-band ESR spectra of the polycrystalline samples were recorded at room temperature using a Varian E-12 X-band spectrometer with 100 kHz modulation frequency in the presence of DPPH as an external standard. TGA/DTG analyses were carried out in the temperature range from 25 to 700 °C under nitrogen employing a Shimadzu DTG 60H thermal analyzer. The experimental conditions were a platinum crucible, nitrogen stream with a 30 mL min^−1^ flow rate and a heating rate of 20 °C min^−1^. The analyses were carried out using SDT-Q600-V20.5-Build-15 at the Institute of Graduate Studies and Research, Alexandria University. The morphologies of the nanosized materials were observed by scanning electron microscopy (SEM) (JEOL-JSM5300) and transmission electron microscopy (TEM) (JTM-1400 Plus) at the E-Microscope Unit, Faculty of Science, Alexandria University. The samples were sonicated in deionized water for 5 min, deposited onto carbon-coated copper mesh and allowed to air-dry before examination.

### Green synthesis of 4,4’-methanedianiline 1

A modified green synthesis, reported by *Bahulayan* et al.^15^, of the ligand 4,4’-methanedianiline **1** was conducted. In an ultrasonic bath at 42 kHz in a water bath, 1 g of the sieved kaolinite powder through two layers of cheesecloth, to ensure particle homogeneity, was added to distilled water (200 ml) followed by successive addition of freshly distilled aniline (9.1 ml, 0.1 mol) and dropwise formaldehyde solution (4.5 ml, 0.05 mol, 34–38%). The mixture was further ultrasonicated for a period of 30 min and stirred using glass rode from time to time. The obtained yellowish-white precipitate was filtered by an air pump, and contaminated crude sample **1** was recrystallized by EtOH from many other byproducts, m.p. 98 °C (Lit.^15^ 96 °C). Unfortunately, the obtained low yield of pure diamine by this method has turned our direction to use commercial **1** in the next step.

### Synthesis of *N,N*'-bis(1-naphthylidene)4,4'-diaminodiphenylmethane 3

2-Hydroxy-1-naphthaldehyde **2** (3 g, 17.44 mmol) dissolved in 15 ml ethanol was added to a warm stirred solution of 4,4'-methanedianiline **1** (1.75 g, 8.8 mmol) in ethanol (15 ml), and the mixture was refluxed for 1 h. The resulting yellow–orange precipitate was filtered, subsequently washed with EtOH and Et_2_O (20 ml each) and then dried in a vacuum oven at 60 °C. Yield: 3.85 g (88%), mp: 275 °C. Anal. Calc. for C_35_H_26_N_2_O_2_ (%) (507); C, 82.98; H, 5.17; N, 5.52. Found; C, 82.53; H, 5.47; N, 6.67. FT-IR ν(cm): 3466 (OH), 1625 (C=N), 1545, 1511, 1350, 1322, 1212, 1140, 970, 827. UV–Vis in DMSO: λ_max_ nm (log ɛ) L mol^−1^ cm^−1^; 274 nm (4.51), 323 nm (4.72), 339 nm (3.74), 388 nm (4.41), 444 nm (4.46), 468 nm (4.28). ^1^H-NMR (500 MHz): d 15.83 (s br, 2H, OH); 9.60 (m, 2H, 2 × H-1); 8.44 (d, 2H J 7.65, Ar H-5, Ar H-5’); 7.88 (d, 2H, J 9.55, Ar H-9, Ar H-9’); 7.74 (d, 2H, J 7.65, Ar H-6, Ar H-6’); 7.55 (d, 2H, J 7.65, Ar H-8, Ar H-8’); 7.50 (m, 4H, Ar H-14, Ar H-14’, Ar-H-16, Ar H-16’); 7.35 (m, 4H, Ar H-13, Ar H-13’, Ar H-17, Ar H-17’); 6.95 (d, 2H, J 9.55 Hz, H-7, H-7’); 6.86 (d, 1H, J 7.65 Hz, Ar H-4); 6.47 (1H, d, J 8.6 Hz, H-4’); 4.86 (s br, 1H, NH–); 4.00 (s; 2H, –CH_2_); 3.75 (s, 2H, -CH_2_). ^13^C-NMR (125 MHz): 171.53 (−C=N), 155.59, 155.36, 142.22, 140.29, 137.32, 137.32, 133.70, 130.11, 129.61, 127.06, 123.26, 122.59, 121.18, 120.98, 120.71, 108.88, 90.68, 88.84, 40.31, 40.14. MS (EI); *m/z* (intensity): 491 M-16 (4), 369 (29.6), 368 (100), 353 (18.5), 339 (11), 314 (19), 283(20), 274 (18), 255 (19.5), 213 (11), 160 (10), 145 (18.5), 131 (13.5), 123 (34.9), 107 (19.5), 105 (35.9), 91 (25.8), 81 (22.5), 69 (10.2), 55 (13.2), 43 (13.3), 29 (2.5).

### Synthesis of metal complexes 5–8 (general method)

A solution of the appropriate metal salt (5.0 mmol) dissolved in EtOH (15 mL) was added to a warm stirred mixture of the ligand (5.0 mmol) and Et_3_N (2.0 mmol) in 1,4-dioxan (15 mL). The mixture was refluxed for 2 h, whereupon the complex was precipitated. It was filtered off, washed with MeOH and Et_2_O and dried in a vacuum oven at 60 °C.

#### Synthesis of zinc complex 5

Following the general procedure given above, a solution of Zn(OAc)_2._2H_2_O (1.09 g, 5.0 mmol) in EtOH was added to the ligand (2.5 g, 5.0 mmol) and Et_3_N (2.0 mmol) in 1,4-dioxan (15 ml). The yellow precipitate was collected in 75% (0.76 g) yield; m.p. > 300 °C, mol. wt. (980.29); Anal. Calc. for C_61_H_48_N_4_O_5_Zn (%); C, 74.58; H, 4.92; N, 5.70; Zn, 6.65. Found %, C, 74.11; H, 4.52; N, 5.23; Zn, 6.60. IR (KBr, u cm^−1^): 3452, 2921, 2852, 1617, 1577, 1502, 1456, 1427, 1391, 1362, 1303, 1249, 1182, 1161, 1143, 1119, 1043, 1017, 984, 872, 553, 495, 449. UV–Vis in DMSO: λmax nm (log ɛ) L mol^−1^ cm^−1^ = 324 nm (3.47), = 427 nm (3.47). ^1^H-NMR (500 MHz, DMSO-d6, total scan = 80): 10.49 (s br, 2H, 2 × OH), 9.58 (m, 2H, 2 × H-1); 8.50 (m, 2H, Ar H-5, Ar H-5’); 7.90 (m, 2H, Ar H-9, Ar H-9’); 7.74 (m, 2H, Ar H-6, Ar H-6’); 7.55 (m, 2H, Ar H-8, Ar H-8’); 7.50 (m, 8H, Ar H-14, Ar H-14’, Ar-H-16, Ar H-16’); 7.35 (m, 8H, Ar H-13, Ar H-13’, Ar H-17, Ar H-17’); 6.95 (d, 2H, J 9.55 Hz, H-7, H-7’); 6.86 (d, 1H, J 7.65 Hz, Ar H-4); 6.47 (1H, d, J 8.6 Hz, H-4’); 4.86 (s br, 2H, 2 × NH-); 4.00 (s; 2H, –CH_2_); 3.75 (s, 2H, –CH_2_), 2.4 (buried) (s, 3H, CH_3_). MS (EI); m/e (intensity): 1178 (3), 1147 (36), 1125 (23), 1076 (13), 998 (9), 947 (9), 906 (24), 815 (6), 729 (8), 671 (2), 611 (10), 555 (4), 481(21), 424 (2), 389 (12), 336 (7), 291 (6), 249 (12), 176 (9), 58 (11), 33 (1). Molar conductance (Ω^−1^ cm^2^ mol^−1^): 30, µ_eff_ (B.M.): diamagnetic.

#### Synthesis of cobalt complex 6

Following the general method described above, a solution of Co(OAc)_2_.H_2_O was prepared (1.24 g, 5.0 mmol) in EtOH was added to the mixture of the prepared ligand (2.5 g, 5.0 mmol) and Et_3_N (2.0 mmol) in 1,4-dioxan (15 ml). The reddish-brown precipitate obtained gave the following physical results: Yield: 79%, m.p. > 300 °C. Mol. wt.; 993; Anal. calc. for C_61_H_50_N_4_O_6_Co (%); C, 73.71; H, 5.07; N, 5.64; Co, 5.93 Found %, C, 74.12; H, 5.54; N, 5.02; Co, 5.88, IR (KBr, μ cm^−1^): 3466, 1617, 1602, 1571, 1534, 1502, 1454, 1425, 1388, 1360, 1302, 1250, 1183, 1162, 1143, 1119, 980, 872, 557, 498, 452. MS (EI); m/z (intensity): 618 M/2 (18.2), 588 (14.4), 517 (2.4), 438 (1.8), 412 (6.5), 393 (18.8), 378 (11.3), 366 (21.8), 333 (10.6), 322 (99.9), 323 (27.1), 283 (7.4), 250 (17.7), 228 (11.8), 209 (13), 180 (16.7), 136 (8.9), 111 (6.2), 95 (11), 89 (13), 69 (7), 59 (40.3), 57 (24.4), 41 (13.9), 30 (37.7), 18 (22.2). UV–Vis in DMSO: λ_max_ nm (log ɛ) L mol^−1^ cm^−1^; 323 nm (3.63), 361 nm (2.69), 442 nm (2.65), 470 nm (2.39), 992 nm (0.61). Molar conductance (Ω^−1^ cm^2^ mol^−1^): 33, µ_eff_ (B.M.): 5.06.

#### Synthesis of copper complex 7

Following the general method, a solution of Cu(OAc)_2_. H_2_O (1.0 g, 5.0 mmol) in EtOH (15 mL) was added to a mixture of the prepared ligand (2.5 g, 5.0 mmol) and Et_3_N (2.0 mmol) in 1,4-dioxin (15 mL). The light brown precipitate was isolated in 82% yield, m.p. > 300 °C, Mol. Wt. 1074.69; Anal. Calc. for C_63_H_54_N_4_O_9_Cu (%); C, 70.41; H, 5.06; N, 5.22; Cu, 5.91. Found %, C, 70.83; H, 3.65; N, 4.27; Cu, 5. 88. IR (KBr, υ cm^-1^): 3465, 1617, 1601, 1577, 1536, 1501, 1456, 1431, 1394, 1365, 1309, 1252, 1185, 1162, 1142, 1094, 1017, 983, 854, 554, 496, 417. UV–Vis in DMSO: λ_max_ nm (log ɛ) L mol^−1^ cm^−1^ = 324 nm (3.60), 424 nm (3.53), 470 nm (3.59). MS [EI, m/e (intensity)]: 1105 (2), 1077 (13), 998 (9), 977 (8), 946 (13), 906 (2), 889 (5), 859 (5), 815 (6), 759 (5), 729 (8), 671 (28), 641 (2), 611 (10), 555 (4), 496 (4), 481 (21), 467 (2), 400 (7), 321 (6), 291 (6), 249 (12), 175 (9), 147 (2), 81 (2), 64 (1), 62 (2), 61 (1), 60 (37), 31 (1). Molar conductance (Ω^−1^cm^2^ mol^−1^): 90, µ_eff_ (B.M.): 2.16.

#### Synthesis of nickel complex 8

Following the general method described above, a solution of NiCl_2_.6H_2_O (1.18 g, 5 mmol) in EtOH was added to a mixture of the ligand (2.5 g, 5.0 mmol) and Et_3_N (2.0 mmol) in 1,4-dioxan. The olive-green precipitate of the complex was obtained with 75% yield, m.p. > 300 °C. The following physical data are collected: mol. wt. 688. Anal. calc. for C_35_H_32_N_2_Cl_2_O_5_Ni (%); C, 60.90; H, 4.67; N, 4.06; Ni, 8.53. Found %, C, 61.07; H, 1.27; N, 4.48; Ni, 8.30. IR (KBr, υ cm^-1^): 3465, 1617, 1601, 1577, 1536, 1501, 1456, 1431, 1394, 1365, 1309, 1252, 1185, 1162, 1142, 1094, 1017, 983, 854, 554, 496, 417. UV–Vis in DMSO: λ_max_ nm (log ɛ) L mol^−1^ cm^−1^; 323 nm (2.60), 340 nm (4.57), 361 nm (4.53), 442 nm (3.11), 471 nm (2.60). MS (EI); m/e (intensity): 977 (12), 946 (20), 919 (23), 906 (37), 889 (8), 859 (9), 831 (8), 815 (100), 801 (46), 759 (8), 729 (12), 671 (4), 611 (15), 555 (6), 496 (6), 481 (32), 467 (3), 400 (11), 349 (5), 321 (9), 291 (9), 263 (8), 249 (18), 175 (13), 121 (3), 58 (46). Molar conductance: (Ω^−1^ cm^2^ mol^−1^): 14, µ_eff_ (B.M.): 4.06.

### Antioxidant assay

#### DPPH (1,1-diphenyl-2-picrylhydrazyl) radical scavenging assay

The oxidative scavenging activity of the ligand and its complexes was evaluated by DPPH (4 mg/100 ml methanol) *Zengin,* et al.^16^. Briefly, equal volumes of different concentrations (0.5, 0.1, 0.2, 0.3, 0.4 and 0.5 mg/mL DMSO) of the tested components were added to DPPH reagent using ascorbic acid as a reference. All the investigated tests were shaken and incubated at room temperature in the dark for 30 min. The decrease in DPPH radicals was evaluated at 490 nm using an ELISA reader. Each test was performed in triplicate. The radical scavenging power was estimated as a percentage of DPPH inhibition using the equation:

$$\% {\text{ inhibition }} = \, [({\text{A \, control}} - {\text{A \, sample}})/{\text{A \, control}}] \, { \times }{ 1}00.$$

#### Nitric oxide scavenging assay

The nitric oxide scavenging activity of the ligand and its complexes was evaluated following a reported procedure *Padmaja,* et al.^17^. Sodium nitroprusside (5 mM, 100 µL) in phosphate buffer (0.1 M; pH 7.6) was added to 50 µL of different concentrations of compounds (50, 100, 300, 400 and 500 µg/mL in DMSO) and incubated for 150 min at 25 °C. The samples were added to 150 µL Griess reagent (1% sulfanilamide and 0.1% naphthyl ethylenediamine hydrochloride in 2.5% orthophosphoric acid) and incubated at 25 °C for 30 min. The absorbance (A) was read at 540 nm using an ELISA reader. The experiment was repeated in triplicate, and ascorbic acid was used as a standard. The scavenged activity of NO was calculated using the equation

$$\% {\text{ inhibition }} = \, [({\text{A \, control}} - {\text{A \, sample}})/{\text{A \, control}}] \, { \times }{ 1}00.$$

#### Reducing power assay

The reducing power capacity of the investigated compounds was evaluated by the method of Oyaizu^18^. Then, 0.5 mL of different concentrations (0.5, 0.1, 0.2, 0.3, 0.4 and 0.5 mg/mL in DMSO) was added to 0.5 mL of sodium phosphate buffer (0.2 M, pH 6.6) and 0.5 mL of 1% potassium ferricyanide. The samples were incubated at 50 °C for 20 min. Then, 0.5 mL trichloroacetic acid (10%) was added and centrifuged at 3000 rpm for 15 min. The supernatant (1.0 mL) was incubated with 1.0 mL H_2_O_2_ and 0.2 mL of 1% ferric chloride for 30 min. The absorbance of the samples was measured at 700 nm. Ascorbic acid was used as a standard. An increase in the absorbance value indicates a higher reducing power.

#### Lipid peroxidation scavenging activity

Lipid peroxidation (LPO) is used as a marker of oxidative stress and tissue damage^19^. LPO was measured as thiobarbituric acid reactive substance (TBARS). Then, 0.5 mL of different concentrations of the tested samples (ligand and its metals 0.1, 0.2, 0.3, 0.4 and 0.5 mg/mL DMSO), distilled water (blank), DMSO (control) or ascorbic acid (reference standard) was added to 0.5 ml liver homogenate (10%, w/v) for 45 min at 37 °C. For peroxidation induction, ferrous sulfate and H_2_O_2_ (0.5 mM and 1 mM, respectively) were added to all tubes except the blank tube and incubated for 30 min at 37 °C. Butylated hydroxyl toluene was added and centrifuged for 10 min at 3000 rpm. One milliliter of the supernatant was mixed with one mL of TCA (20%) and centrifuged at 3000 rpm for 15 min. Then, 0.5 mL of 0.7% thiobarbituric acid (TBA) was added to 1 mL of the supernatant and heated for 1 h at 100 °C in a boiling water bath. The color development was evaluated at 532 nm against the blank. The antioxidant activity of the tested compounds was assessed as the percentage inhibition of LPO in liver homogenate as follows:

$$\% {\text{ Inhibition \, of \, lipid \, peroxidation }} = \, [\left( {{\text{A \, control }} - {\text{ A \, sample}}} \right)/{\text{A \, control}}] \, { \times }{ 1}00.$$

#### Determination of total antioxidants

The phosphomolybdate assay was used to evaluate the total antioxidant capacity of the ligand and its metal complexes. The assay is based on the reduction of molybdate(VI) to molybdate(V) by the samples and subsequent formation of a green phosphate Mo(V) complex at low pH using ascorbic acid as a standard. One hundred microliters of 0.5 mg/ml samples (Schiff base ligand and its metal complexes) or various concentrations of ascorbic acid was added to 1.9 ml antioxidant reagent (0.6 M H_2_SO_4_, 28 mM sodium dihydrogen phosphate and 4 mM ammonium molybdate) and incubated at 95 °C for 90 min. The samples were then cooled, and the absorbance was measured at 695 nm. Ascorbic acid was utilized as a reference standard^20,21^. The ascorbic acid standard curve was plotted to calculate the total antioxidant content in one mg.

#### Determination of membrane stabilization activities

Anti-inflammatory activities were evaluated through the antihemolytic membrane stabilization effect of the ligand and its metal complexes using a red blood cell (RBC) membrane stabilization method^22^. Fresh whole blood samples were collected in anticoagulant tubes from a healthy volunteer who was not treated with any nonsteroidal anti-inflammatory drugs (NSAIDs) for 2 weeks before the experiment. Erythrocytes were extracted by centrifugation at 3000 rpm for 5 min. The anticoagulated cells were washed 3 times with 0.9% saline and centrifuged at 3000 rpm for 10 min. An RBC suspension (40% v/v) was prepared using isotonic saline phosphate buffer (0.15 M NaCl and 10 mM sodium phosphate buffer, pH 7.4). One milliliter of phosphate buffer (0.15 M, pH 7.4) was added to one mL of various concentrations of the tested compounds (0.05, 0.1, 0.2, 0.3, 0.4 and 0.5 mg/mL DMSO) and 2 mL sodium chloride solution (0.25%). In the control tube, one distilled water was used instead of the tested compounds. Different concentrations of nonsteroidal anti-inflammatory drug (diclofenac potassium) were used as standards. Then, 100 µL of RBC suspension was added to each tube and incubated at room temperature for 60 min. After incubation, the samples were centrifuged for 10 min at 3000 rpm. The absorbance of the supernatant was detected at 540 nm using an ELISA reader. The inhibition of hemolysis in the samples was evaluated according to the equation:

$$\% {\text{ Inhibition \, of \, hemolysis}}\, = \,\left[ {\left( {{\text{A \, control}} - {\text{A \, sample}}} \right)/{\text{A \, control}}} \right]\, \times \,{1}00.$$

#### Determination of AChE inhibition

Acetylcholinesterase (AChE) activity was evaluated according to a previously reported method^23^. Briefly, 10 μL of different concentrations of the tested compounds (test) or organic solvent (control) was added to 130 μL of phosphate buffer (pH 7.4, 0.1 M) and 20 μL of brain homogenate and then incubated at 37 °C for 45 min. Then, 5 μL of acetylcholine iodide (75 mM) was added and incubated for 15 min at 37 °C. After incubation, 60 mL of 0.32 mM DTNB was added. The absorbance was recorded at 405 nm after 5 min using donepezil (AChEI) as a standard. The percent inhibition of AChE was calculated as follows:

$$\% {\text{inhibition \, of \, lipid \, peroxidation}}\, = \,[\left( {{\text{A \, control}}{-\!\!-}{\text{A \, sample}}} \right)/{\text{A \, control}}]\, \times \,{1}00.$$

### Antimicrobial activity

The ligand and its metal(II) complexes were evaluated for antimicrobial activity against two strains, gram-positive bacteria (*Staphylococcus* *aureus* and *Staphylococcus* *faecalis*), gram-negative bacteria (*Escherichia* *coli* and *Pseudomonas* *aeruginosa*) and pathogenic fungi (*Candida* *albicans*), using DMSO as a negative control. Tetracycline was used as a positive standard for antibacterial activities, and amphotericin B was used as a positive standard for antifungal activities. The antimicrobial activity of the tested samples was determined using a modified Kirby-Bauer disc diffusion method^24^. All the synthesized compounds were dissolved to prepare a stock solution of 1 mg/mL using DMSO. Stock solution was aseptically transferred, and two-fold diluted to have solutions of different concentrations. The antibacterial and antifungal activities of test compounds were done by filter paper disc method^21^ and the activities were determined by measuring the diameters of the inhibition zone (mm). Media with DMF was set up as a control. All cultures were routinely maintained on NA (nutrient agar) and incubated at 37 °C. The inoculums of bacteria were performed by growing the culture in NA broth at 37 °C for overnight. Approximately, 0.1 mL of diluted bacterial or fungal culture suspension was spread with the help of spreader on NA plates uniformly. Solutions of the tested compounds and reference drugs were prepared by dissolving 10 mg of the compound in 10 mL DMF. A 100 μL volume of each sample was pipetted into a hole (depth 3 mm) made in the center of the agar. Sterile 8 mm discs (Himedia Pvt. Ltd.) were impregnated with test compounds. The disc was placed onto the plate. Each plate had one control disc impregnated with solvent. The plates were incubated at 37 °C for 18–48 h. Standard discs of Tetracycline (Antibacterial agent; 10 μg/disc) and Amphotericin B (Antifungal agent; 10 μg/disc) served as positive controls for antimicrobial activity while filter discs impregnated with 10 µL of solvent DMSO were used as a negative control. All the experiments were performed at least in triplicate and the outcomes were averaged.

## Results and discussions

### Green synthesis of 4,4’-methylenedianiline 1

**Figure 1:**
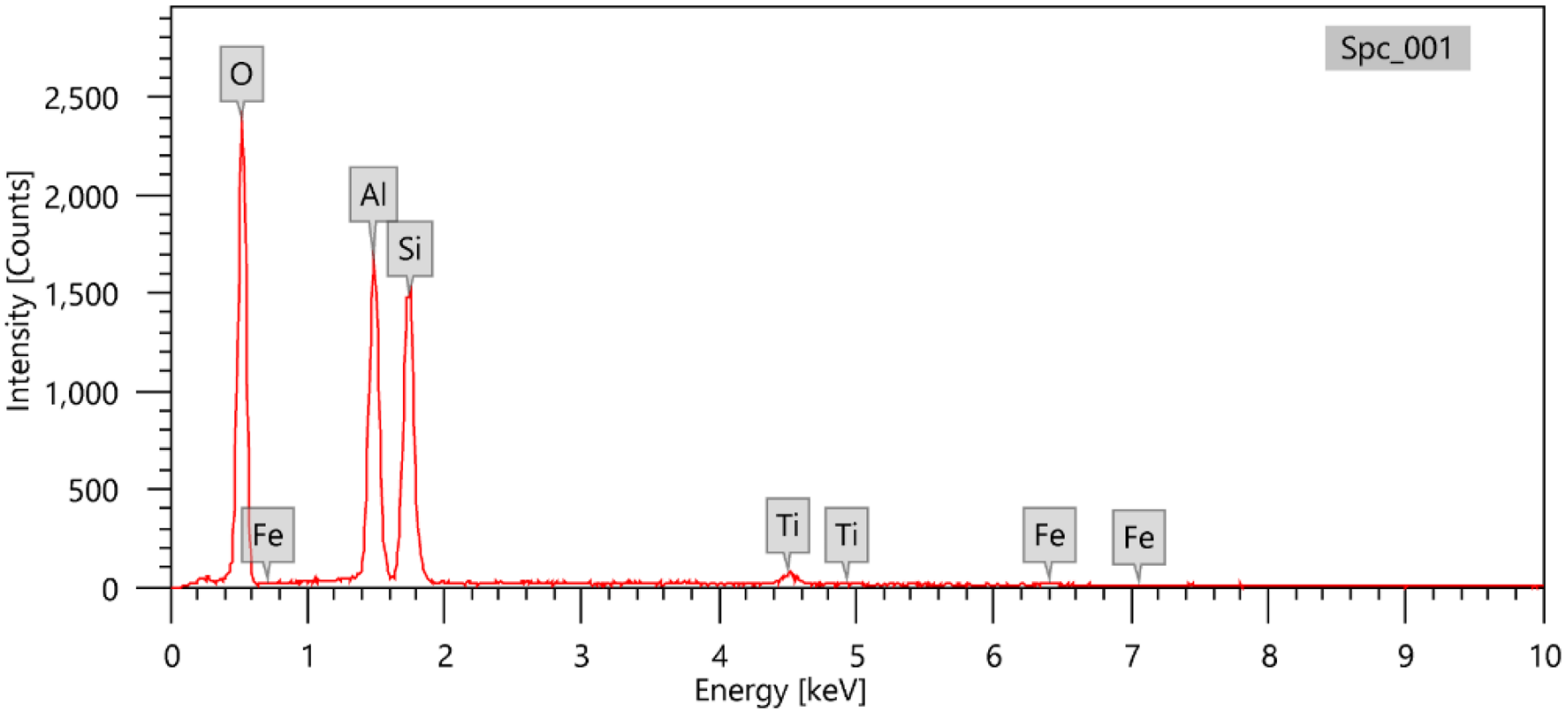
Energy dispersive X-ray (EDX) analysis of the kaolinitic clay sample.

A modified green synthesis of the ligand 4,4’-methanedianiline was conducted^15^. The reaction of aniline (2 equivalents) and formaldehyde solution (34–38%, 1 equivalent) mediated by natural kaolinite was performed in an ultrasonic bath at 42 kHz in a water bath for 20 min. Kaolinite is a 1:1 layer phyllosilicate clay mineral, and its chemical formula is Si_2_Al_2_O_5_(OH)_4,_ Al_2_O_5_Si_2_.2H_2_O or Al_2_O_3_.2Si_4_.2H_2_O)^2^ along with other minor metal oxide impurities. Its structure possesses a sheet of octahedral Al(OH)_4_ bonded via H-bonds to a sheet of combined tetrahedral SiO_4_. Notably, no reaction occurs using other carbonate-containing clays, such as limestone or dolomite, as catalysts. According to the EDX analysis shown in Fig. 1, the obtained kaolinitic clay has the following composition (mass %): Al_2_O_3_ (40.62 ± 0.51), SiO_2_ (55.32 ± 0.71), TiO_2_ (2.86 ± 0.17) and FeO (1.20 ± 0.12).

### Reaction mechanism

**Figure 2:**
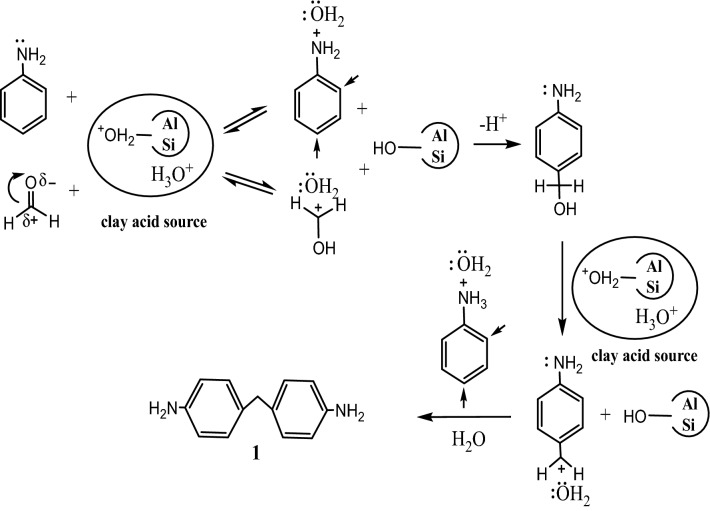
Proposed mechanism of the kaolinite-catalyzed synthesis of diamine 1.

The kaolinite particles possess oppositely charged surface regions in aqueous media due to bonding of the silicate oxygen with OH’s octahedral sheet. The protonation pH (≅ 7)/deprotonation (> 7) in the aqueous phase develops charges on the edges and faces of the octahedral-oxygen sheets, causing surface charge heterogeneity. In aqueous medium, kaolinite acidic sources developed by protonation of aluminol Al–OH sites (Al–OH + H^+^  → Al-OH_2_^+^), silanol (Si–OH) sites (Si–OH + H^+^  → Si-OH_2_^+^) and coordinated water molecules formed via a prototropy process (proton migration) of two hydroxyl units (OH^−^ ↔ H^+^  + O^2−^, H^+^  + OH^−^ ↔ H_2_O, H_2_O + H^+^  → H_3_O^+^)^25^. Figure 2 depicts the suggested reaction mechanism for diamine **1** formation. In aqueous medium, the kaolinite acidic sources are different from both the basic aniline nitrogen and the partially negatively charged oxygen of formaldehyde. In the former case, the reaction would give the Al-OH_2_^+^/Si-OH_2_^+^ sites, while the latter would give the speculated carbocation, releasing hydrated Al–OH and Si–OH sites in both cases. Electrophilic attack of the aldehyde carbocation at the para position of a hydrated anilinium molecule would give the intermediate (4-aminophenyl) methanol. The reaction of the kaolinite acidic sources with the alcoholic intermediate would give the corresponding 4-aminobenzyl cation and release the hydrated Al–OH sites. Reaction of the carbocation with a second hydrated anilinium molecule would give the expected target hydrated methylenedianiline. Notably, in the presence of such a hydrated and variable acidic source medium, another reaction of the acidic-trapped electron-rich amino-nitrogen center in either step was not expected.

### Synthesis of *N,N*'-bis(1-naphthylidene)4,4'-diaminodiphenylmethane 3

**Figure 3:**
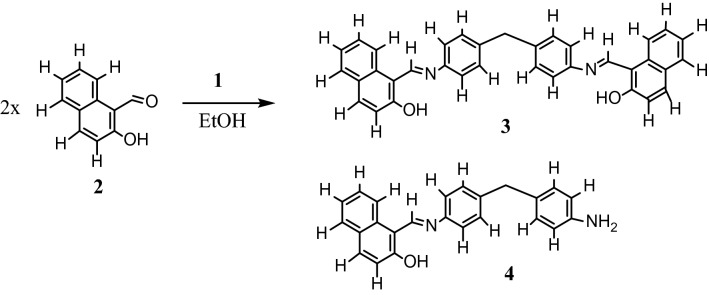
Chemical synthesis of the ligand mixture (3,4).

Condensation of the synthesized methanedianiline **1** with two equivalents of the commercial 2-hydroxy-1-naphthaldehyde **2** in boiling EtOH for 1 h produced a (1:1) mixture of the targeted bis-imine **3** and the Schiff base **4** as a homogenous yellow‒orange solid, Fig. 3. Several trials to separate these two analogs by recrystallization and/or chromatography failed.

IR analysis of ligand mixtures **3** and **4** exhibited a broad band at υ 3466 cm^−1^ corresponding to the phenolic OH groups, a strong band at υ 1625 cm^−1^ due to the imine bonds CH=N– and bending bands at υ 1545, 1511, 1350, 1322, 1212, 1140, 970, and 827 cm^−1^. The ^1^H-NMR spectrum exhibited a broad singlet signal at δ 15.83 ppm (s br, 2H, OH), a multiplet signal at δ 9.60 ppm corresponding to the two imine protons (H-1), a doublet signal at δ 8.44 ppm (J 7.65 Hz) corresponds to two protons (Ar H-5, Ar H-5’), a doublet signal at δ 7.88 ppm (J 9.55 Hz) due to two protons (Ar H-9, Ar H-9’), a doublet signal at δ 7.74 (J 7.65 Hz) due to two protons (Ar H-6, Ar H-6’), a doublet signal at δ 7.55 ppm (J 7.65 Hz) due to two protons (Ar H-8, Ar H-8’), a multiplet signal at δ 7.50 ppm for four protons (Ar H-14, Ar H-14’, Ar–H-16, Ar H-16’), a multiplet signal at δ 7.35 ppm due to four protons (Ar H-13, Ar H-13’, Ar H-17, Ar H-17’), a doublet signal at δ 6.95 ppm (J 9.55 Hz) corresponds to two protons (H-7, H-7’), a doublet signal at δ 6.86 ppm (J 7.65 Hz) due to one proton (Ar H-4), a doublet signal at δ 6.47 ppm (J 8.6 Hz) due to one proton (Ar H-4’), a broad singlet signal at δ 4.86 ppm due to two protons (NH_2_); a singlet signal at δ 4.00 ppm due to two aliphatic protons) CH_2_); and a singlet signal at δ 3.75 ppm due to two aliphatic protons 3.75 (CH_2_). The ^13^C-NMR spectrum exhibited three imines (–C=N) with distinguished signals at δ 171.53 ppm, 155.59 ppm, and 155.36 ppm. Other aromatic carbons resonated at δ 142.22 ppm, δ 140.29 ppm, δ 137.32 ppm, δ 137.32 ppm, δ 133.70 ppm, δ 130.11 ppm, δ 129.61 ppm, δ 127.06 ppm, δ 123.26 ppm, δ 122.59 ppm, δ 121.18 ppm, δ 120.98 ppm, δ 120.71 ppm and δ 108.88 ppm. The carbon atoms of the two aliphatic (-CH_2_-) atoms resonated at δ 40.31 ppm and δ 40.14 ppm. Electron ionization mass spectrometry analysis showed a fragment peak at *m/z* 491 corresponding to (**3**, M^+^-OH), and the rest of the fragmentation peaks appeared at m/z 369, 368 (base peak), 353 (**4**, M^+^  + 1), 339, 314, 283, 274, 255, 213, 160, 145, 131, 123, 107, 105, 91, 81, 69, 55, 43, and 29. Anal. Calc. for C_59_H_46_N_4_O_3_ (%) (858.36); C, 82.49; H, 5.40; N, 6.52. Found; C, 82.53; H, 5.47; N, 6.67.

**Figure 4:**
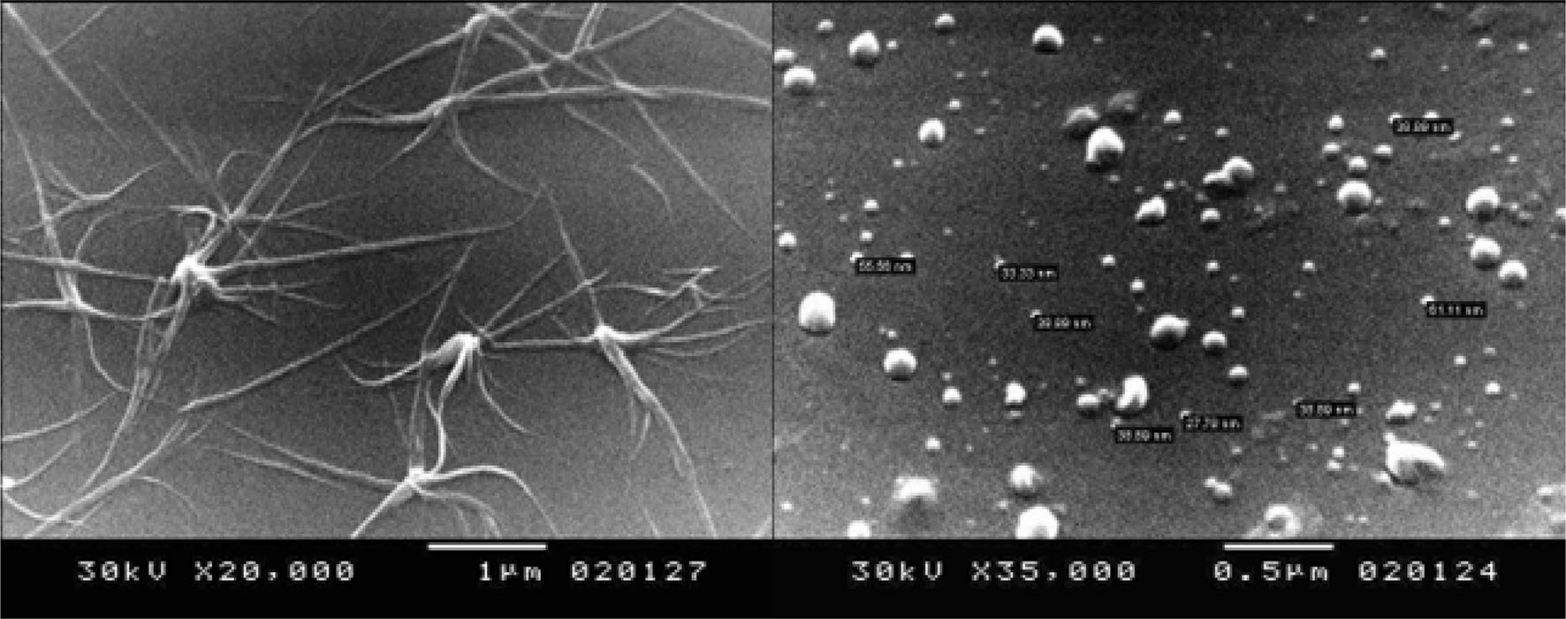
SEM images of the mixed ligand 3.

A scanning electron microscopy (SEM) photograph of the ligand mixture, Fig. 4, indicated that the aggregated particles that appeared as an Octopus-like morphology were self-assembled from spherical nanosized particles with an average diameter of 40 nm. Such morphology could be attributed to colloidal self-assembly, which relied solely on particle surface chemistry, based on both the hydrophobic–hydrophilic interaction mechanism and the presence of water^25^.

### Synthesis of metal complexes 5–8

**Figure 5:**
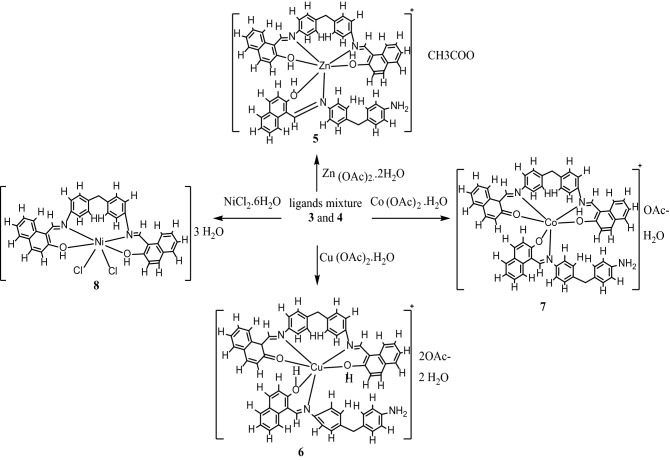
Chemical synthesis of metal complexes 5–8.

An ethanolic solution of the appropriate metal salt, namely, Co(OAc)_2_. H_2_O, NiCl_2_.6H_2_O, Cu(OAc)_2_. H_2_O and Zn(OAc)_2._ 2H_2_O was heated with a mixture of the ligand and Et_3_N in 1,4-dioxan for 2 h, and the corresponding formed complex (**5**–**8**) was filtered and worked up (Fig. 5). The synthesized metal (II) complexes are air-stable at room temperature, insoluble in water, chloroform, and most organic solvents but freely soluble in DMSO and DMF. The observed molar conductivity values for the 1.00 × 10^–3^ M DMSO solution at 25 ± 1 °C for zinc and cobalt complexes are found to be 30 S⋅cm^2^⋅mol^−1^ and 33 S⋅cm^2^⋅mol^−1^, respectively, indicating a 1:1 electrolyte. The molar conductance was calculated using the equation: Λm = K/C, where K = specific Conductivity, C = concentration in mole per liter. The molar conductivity value of 90 S⋅cm^2^⋅mol^−1^ for the copper complex revealed a 1:2 electrolyte. However, the detected lower molar conductivity value of 14 S⋅cm^2^⋅mol^-^1 for the nickel complex estimated its nonelectrolyte nature^26^. The IR spectra of the ligand and its complexes showed broad bands in the range *υ* 3466–3452 cm^−1^ assignable to the phenolic OH, the nonacoordinate NH_2_’s or water molecules associated with the complexes. The *υ* C=N_str_ of the ligand appeared at *υ* 1625 cm^−1^. This band was slightly shifted to a lower wavenumber at *υ* 1617 cm^−1^ in all metal complexes, confirming the participation of the azomethine' nitrogen in chelation. The complexes of copper **6**, cobalt **7** and nickel **8** each showed strong bands at *υ* 1602 cm^−1^, *υ* 1601 cm^−1^ and *υ* 1601 cm^−1^, respectively, attributed to the keto group. However, in the case of zinc complex **5**, u_C=O_ was not observed, indicating the participation of the azomethine’s nitrogen in chelation, as suggested in the structure. Moreover, the bands at approximately u 1534–u 1536 cm^−1^ found only in complexes **6**–**8** were attributed to the *υ* NH–C=C–C=O_str_ tautomer. The prepared ligand band at *υ* 1322 cm^−1^ assigned to *υ* (C–O) was shifted to a lower wavenumber ranging in all complexes at *υ* 1302–1309 cm^−1^. Bands associated with M–N and M–O bonds were assigned, respectively, at u 449 cm^−1^, *υ* 553 cm^−1^ for complex **5**, u 417 cm^−1^, u 554 cm^−1^ for complex **6**, at *υ* 452 cm^−1^, *υ* 557 cm^−1^ for complex **7** and *υ* 417 cm^−1^, *υ* 554 cm^−1^ for complex **8**^27^.

### Magnetic moment and electronic absorption spectra

Magnetic susceptibility measurements were carried out on the complexes in the solid-state at room temperature. The mass susceptibility, χ_g_, was calculated using the equation: χ_g_ = *C*_bal_.*l*.(*R* − *R*_0_)/10^9^ m, where *C*_bal_ is the balance calibration constant (= 2.086); *l* is the sample length (cm); *m* is the sample mass (gm); R is the reading for tube plus sample; *R*_0_ is the empty tube. The magnetic μ_eff_ moment was calculated using the equation: μ_eff_ = √χ_g_ × MWt × t (°C)^28^. The UV–Vis spectra of the complexes were recorded in DMSO solution. The electronic spectrum of the prepared ligand in DMSO displayed intense bands at 274, 323 (sh), 339 (sh), 388 (sh), 444 and 468 nm. The former two bands could be assigned to π–π* transitions, while the latter could be assigned to charge transfer transitions. The effective magnetic moment of zero for zinc complex 5 confirms its diamagnetic nature, while the single high-intensity band at 468 nm could be assigned to charge transfer rather than a d–d transition. The Zn(II) complex is found to be diamagnetic, as expected for the d10 configuration, and an octahedral geometry is proposed for this complex. The electronic spectrum of copper complex **7** exhibited bands at 690 and 550 nm, which may be assigned to ^2^B_1g_ → ^2^E_g_ and ^2^B_1g_ → ^2^A_1g_, respectively^29^. These bands favored distorted octahedral geometry around the Cu(II) ion and were supported by the magnetic moment value of 2.16 BM^30^. Moreover, the meff value of nickel complex 8 is 4.06 B.M. which agrees with the reported values for octahedral, tetrahedral, or high spin five coordinate nickel(II) complexes. The electronic spectrum of nickel complex 8 in DMSO shows absorption bands at 323, 340, 361, 442, and 471 nm. Transitions assigned to ^3^A_2g_ → ^3^T_1g_ (F) and ^3^A_2g_ (F) → ^3^T_1g_ (P) are hidden by the very intense charge transfer and ligand absorption bands. The μ_eff_ value of cobalt(II) complex 6 is 5.06 B.M., suggesting an octahedral environment for Co(II)^31,32^. The electronic spectrum of the cobalt complex in DMSO shows absorption bands at 323, 361, 442, 470, and 992 nm. The latter d–d transition in the visible region is assigned to ^4^T_1g_ (F) → ^4^A_2g_(P)^33^.

### Electron paramagnetic resonance spectra

**Figure 6:**
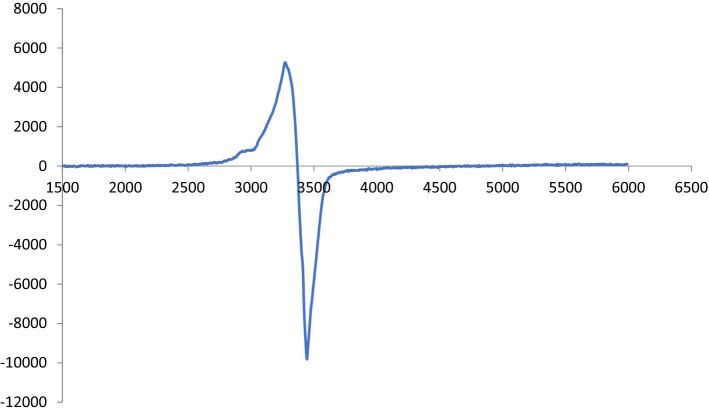
X-band EPR spectrum of Cu(II) complex 7 at room temperature.

The X-band EPR spectrum of Cu(II) complex 7 at room temperature (Fig. 6) is anisotropic with a parallel and perpendicular spin being assignable. The copper complex exhibited a g∥ value of 2.373 and g⊥ value of 2.077. The axial pattern with g∥ > g⊥. implying that the unpaired electron resides in d_x2–y2_ with 2B1 g as the ground state. This spectral feature is consistent with the octahedral arrangement around Cu(II)^34^. The complex exhibited a value of g_av_ = 2.17, and deviation from g_av_ suggested the high covalence property of the complexes with distorted symmetry. The parameter G was found to be higher than 4 (G = 4.84), indicating negligible exchange interaction of Cu-Cu in the complex^35^. Thus, based on the EPR analysis of the investigated Cu(II) complex 7, the greater g_av_ value indicated the presence of Cu–O and Cu–N bonds in these chelates^36^.

### Thermal analysis

The thermogravimetric (TGA) and derivative thermogravimetric (DTG) plots of the prepared ligand and its investigated metal complexes **5**–**8** in the range of 25–700 °C under N_2_ and their stepwise thermal postulated degradation data are compiled in Table 1. The TGA/DTG curve of the ligand exhibited three successive decompositions at 200 °C, 375 °C and 515 °C, attributed to the elimination of water molecules, cleavage of the diphenyl-methylene linkage and further elimination of the phenyl moiety leaving C_11_H_9_ as a residue. The TGA profile of zinc complex **5** showed two decomposition steps; the first process at 150 °C may be related to dehydration of water molecules and elimination of nonacoordinate -OAc groups and NH_2_ groups (Calc. 11.32%; Found 11.96%). The second decomposition step at 450 °C may correspond to the cleavage of the diphenyl-methylene fragments (Calc 34.28%; Found 33.59%), leaving mass residue assigned as C_35_H_21_N_2_Zn (Calc. 54.48%; Found 51.78%). The thermal profile of complex **6** exhibited three significant thermal events within the temperature range of 25–700 °C. The first revealed an exothermic peak with a mass loss of 9.19% (Calc. 9.36%) at 216 °C, corresponding to the elimination of adsorbed water, the coordinated OAc group and the NH_2_ group. The second decomposition step occurred at 450 °C (mass loss not determined), and the major fragmentation step took place up to 690 °C with a mass loss of 45.94% (Calc. 46.12%) attributed to the loss of C_35_H_24_N. The residual mass could be assigned to C_24_H_20_N_22_O_3_ Calc 44.40%; Found 44.48%). Decomposition of copper complex **7** proceeded in one main broad step occurring in the temperature range 311–690 °C with a total mass loss of 54.63%, attributed to the loss of the hydrated bis-ligand moiety leaving a remaining mass residue C_26_H_21_N_2_O_2_Cu (Calc. 43.60%; Found 42.06%). The thermal profile of complex **8** exhibited four thermal fragmentation steps within the temperature range of 25–700 °C. The first revealed an exothermic peak with a mass loss of 8.63% (Calc. 7.97%) at 200 °C, corresponding to the dehydration of adsorbed water molecules. The successive second and third decomposition steps occurred at 460 °C and 550 °C (mass loss not determined), and the major fragmentation step took place at 690 °C with a mass loss of 55.93% (Calc. 55.53%) attributed to the loss of the dehydroxylated bis-ligand moiety. The residual mass was 34.83% assigned to C_10_H_8_ClO_2_Ni (Calc. 34.25%).

**Table 1:** Thermal analysis of mixed ligand 3 and their metal(II) complexes **5**–**8**.

Compound	Molar mass	TGA range °C	DTG max	% Weight loss (calc)	Residue	Assignment
Prepared ligand = ligands mixture	858	23–275	200	4.30 (4.19)	16.05 (16. 90)	2H_2_O
		275–450	375	53.24 (53.37)		C_30_H_26_N_4_O
		450–700	515	25.57 (25.99)		C_18_H_7_
						Res.: C_11_H_9_
**5**	980	23–225	150	11.96 (11.32)	51.78 (54.48)	-OAc, 2H_2_O, NH_2_
		375–700	450	33.59 (34.28)		C_24_H_18_NO
						Res.: C_35_H_21_N_2_Zn
**6**	993	216	110	9.19 (9.36)	44.48 (44.40)	-OAc, H_2_O, NH_2_
		450	375	45.94 (46.12)		C_35_H_24_N
		690	450			Res.:C_24_H_20_N_2_O_3_Co
**7**	1074	311	380	3.31 (3.43)	42.06 (43.60)	2H_2_O
		690	525	54.63 (54.09)		C_37_H_29_N_2_O_5_
						Res.: C_26_H_21_N_2_O_2_Cu
**8**	688	200	39	8.635 (7.80)	34.83 (36.90)	3H_2_O
		450	455	ND		C_25_H_18_N_2_Cl
		550	555	ND		Res.: C_10_H_8_ClO_2_Ni
		690		55.93 (55.45)		

### Antimicrobial activity

The prepared ligand and its metal (II) complexes were evaluated for antimicrobial activity against two strains, gram-positive bacteria (*S*. *aureus* and *S*. *faecalis*), gram-negative bacteria (*E*. *coli* and *P*. *aeruginosa*) and pathogenic fungi (*C*. *albicans*), using DMSO as a negative control. Tetracycline was used as a positive standard for antibacterial activities, and amphotericin B was used as a positive standard for antifungal activities. The obtained antimicrobial results are presented in Table 2. The data showed that the prepared ligand and the Co(II) complex have no efficacy against these microbes, while the Cu(II) complex showed reasonable activity against only E. coli. Both Zn(II) and Ni(II) complexes exhibited comparable moderate activities towards all studied microbes. Notably, the octahedral Ni(II) complex exhibited a sole moderate antifungal activity, whereas all other samples had no activities towards the studied fungus. The increased activity of the metal chelates can be explained based on the overtone concept and chelation theory^37^, in which metal chelates deactivate various cellular enzymes that play a vital role in various metabolic pathways of these microorganisms. Nevertheless, the variation in the activity of different metal complexes against different microorganisms depends on the impermeability of the cells of the microbes or differences in ribosomes in microbial cells^38^. The higher antimicrobial activity of the nickel(II) complex relative to other metal complexes may be due to its structure, where the octahedral nickel(II) complex is formed from the coordination of the bis ligand **3** only to the nickel(II) center, as shown in Fig. 5. However, the other investigated octahedral metal(II) complexes are formed from both ligand mixtures, and this may form nickel(II)-ligand bonds stronger than other M(II)–ligand bonds, which in turn increases the lipophilic character of nickel(II) complexes and favors permeation through the microbial cell membrane, thus destroying them more aggressively. In conclusion, the less bulky octahedral nickel(II) complex enhances its rate of uptake/entrance and thus increases its antimicrobial activity.

**Table 2:** Antimicrobial activity of the ligand and its metal(II) complexes.

Sample	Inhibition zone diameter (mm/mg sample)				
	*E*. *coli*	*P*. *aeruginosa*	*S*. *aureus*	*S*. *faecalis*	*C*. *albicans*
Control: DMSO	Nil	Nil	Nil	Nil	Nil
Tetracycline Antibacterial agent	32	33	32	33	–
Amphotericin B Antifungal agent		–	–	–	20
Ligand	Nil	Nil	Nil	Nil	Nil
Cobalt complex	Nil	Nil	Nil	Nil	Nil
Copper complex	12	Nil	Nil	Nil	Nil
Zinc complex	12	13	14	11	Nil
Nickel complex	11	13	12	12	13

Nil, zero inhibition.

### Antioxidant activities

Oxidative stress is a result of a free radical/antioxidant imbalance that negatively deregulates a cascade of cellular reactions leading to tissue injury and various pathological disorders. This imbalance can damage vital biomolecules, such as carbohydrates, lipids, proteins, nucleic acids and DNA, accelerating cellular death as the basis of several pathological consequences^39,40^. Antioxidants have a crucial role in the human body to slow oxidative stress and its harmful effects. Antioxidant compounds can scavenge of free radicals and lipid peroxidation repairing the cell damage and retarding the progress of various diseases induced by oxidative damage^41^. In this study, the antioxidant capacities of the ligand and its metal complexes were measured using vitamin C as a standard to evaluate the antioxidant properties of these synthesized compounds.

#### DPPH radical scavenging activity

**Figure 7:**
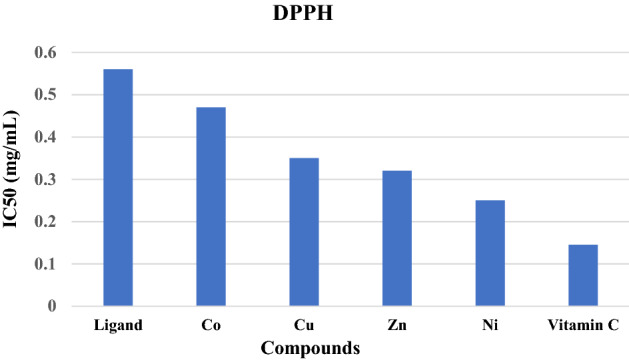
DPPH IC50 values for vitamin C, the ligand, and its metal complexes.

The percentage of the radical scavenging activity of the ligand and its metal complexes (Cu, Zn, Ni, Co) were evaluated using vitamin C as a standard (Table 3, Fig. 7). Free ligand showed the lowest antioxidant activity when compared to all metal complexes at different concentrations (0.05, 0.1, 0.2, 0.3, 0.4 and 0.5 mg/mL DMSO). Complexation with metals significantly (p < 0.01) enhanced the free radical scavenging capacity. The nickel complex showed the highest DPPH activity (IC50 values 0.25 mg/mL), followed by zinc (IC50 values 0.32 mg/mL), copper (IC50 values 0.35 mg/mL) and cobalt complex (IC50 values; 0.45 mg/mL). The free radical scavenging activity of these complexes was significantly lower than (p < 0.01) that of vitamin C (IC50 value 0.145 mg/mL). The obtained data demonstrated that the antioxidant activity of the investigated compounds against DPPH radicals was concentration dependent, in agreement with the reported results that showed the antioxidant capacity of the Schiff base ligand, and their complexes increased with the concentration of the compounds^42,43^. The oxidant activity was reversed by these Schiff base complexes due to their ability to reduce the radicals, preventing their harmful effect. The capacity of antioxidants depends on their way to neutralize the radicals that are produced in biological systems by donating an electron^44,45^.

**Table 3:** The effect of the ligand and its complexes cobalt (Co), copper (Cu), zinc (Zn) and nickel (Ni) on the antioxidant DPPH at different concentrations (0.05–0.5 mg/mL).

Concentration	Parameters					
	Ligand	CO	Cu	Zn	Ni	Standard
0.05	11.5 ± 0.95	14.8 ± 1.15	23.03 ± 0.74	26.63 ± 0.86	29.73 ± 0.45	38.1 ± 1.25
0.1	17.53 ± 0.85	23.5 ± 1.11	30.23 ± 0.49	32.5 ± 0.66	37.1 ± 1.18	46.87 ± 0.44
0.2	25.33 ± 0.81	30.3 ± 0.81	38.07 ± 0.61	39.47 ± 0.6	46.67 ± 0.86	55.6 ± 0.7
0.3	31.13 ± 0.65	37.83 ± 0.61	44.91 ± 0.66	47.83 ± 0.83	52.67 ± 1.21	66.93 ± 1.46
0.4	38.83 ± 0.81	45.23 ± 1.12	54.0 ± 0.95	56.53 ± 0.71	64.87 ± 0.73	79.27 ± 0.76
0.5	44.43 ± 0.70	50.23 ± 0.38	62.40 ± 1.21	65.43 ± 0.85	74.53 ± 1.1	88.73 ± 1.46

#### Nitric oxide scavenging activities

**Figure 8:**
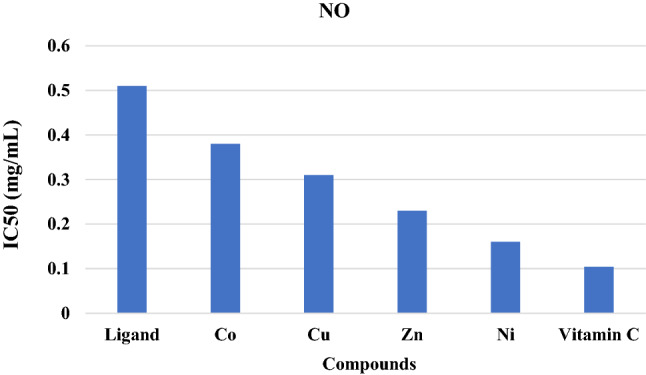
Nitric oxide (NO) IC50 value for vitamin C, ligand, and its metal complexes.

Nitric oxide (NO) plays an essential bioregulatory role in several biochemical processes, such as the immune response and neural signal transmission. However, the excessive production of NO is cytotoxic and induces various physiopathological conditions, including cancer. It reacts with superoxide radicals to form highly reactive peroxynitrite anions, which can induce lipid peroxidation and interfere with cellular signaling, causing damage to cellular proteins^46,47^. Additionally, NO is involved in apoptosis induction, cell cycle interruption, DNA disruption and protein modification^47^. The NO inhibitory effect of the ligand and its metal complexes was detected using ascorbic acid as a standard (Table 4 and Fig. 8). The scavenging effect of the metal complexes (Co, Cu, Zn, Ni) was more significant (p < 0.01) than that of the free ligand. The inhibition ratio of the free ligand and its complexes was concentration dependent, in agreement with the literature^44^, where the percentage of NO suppression increased with increasing sample concentration. Ascorbic acid showed the highest oxidant scavenging ability compared to all the synthetic compounds at p < 0.0. The nickel complex showed the most effective metal (IC50 = 0.16 mg/mL), while Cobalt exhibited the lowest effect (IC50 = 0.38 mg/m). The complexes may have the ability to counteract the harmful effect of NO formation by repairing the damaging effects of excessive NO generation, which may be important for protecting human health.

**Table 4:** The effect of the ligand and its complexes on the antioxidant nitric acid (NO) at different concentrations (0.05–0. 5 mg/mL).

Concentration	Parameters					
	Ligand	CO	Cu	Zn	Ni	Standard
0.05	15.8 ± 0.78	25.5 ± 0.80	29.3 ± 0.95	33.03 ± 0.64	37.27 ± 0.91	42.13 ± 0.91
0.1	21.37 ± 1.38	30.37 ± 0.7	35.4 ± 0.96	39.37 ± 0.81	47.0 ± 1.44	51.27 ± 0.44
0.2	27.13 ± 1.58	35.9 ± 1.54	44.77 ± 1.36	48.23 ± 0.96	55.4 ± 0.98	61.57 ± 2.1
0.3	37.27 ± 0.97	41.87 ± 1.46	48.73 ± 0.45	57.43 ± 1.36	63.4 ± 1.04	68.53 ± 1.01
0.4	42.7 ± 1.78	51.07 ± 1.4	55.0 ± 1.64	64.53 ± 1.26	68.33 ± 1.0	78.53 ± 0.74
0.5	47.65 ± 1.49	60.07 ± 1.47	65.07 ± 1.4	69.47 ± 0.76	74.57 ± 1.9	85.17 ± 1.35

#### Lipid peroxidation (TBARS)

**Figure 9:**
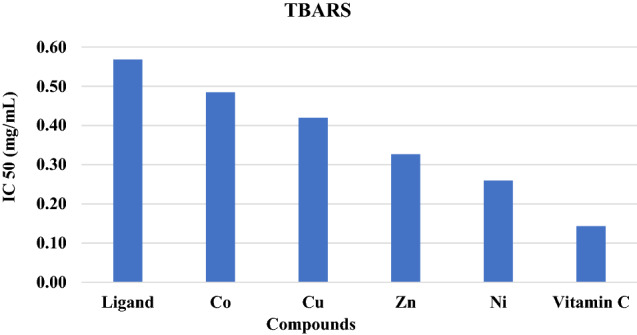
TBARS IC50 values for vitamin C, its ligand, and its metal complexes.

Lipids play an essential role in cell membrane structure and function. All body biochemical, immunological, and physiological processes are associated with structural and functional biological membranes. The peroxidative reaction of the lipid component of cellular membranes by free radicals results in lipid peroxidation^48,49^. LPO has a serious role in triggering many pathological disorders by degrading cellular membrane integrity and leakage of cytoplasmic components. Free radical scavenging is a common system that inhibits lipid peroxidation in the body by antioxidants^50^. The TBARS assay is the most widely used method for determining the lipid peroxidation process. MDA is produced by the degradation of polyunsaturated fatty acids, which react with TBA^51^. A TBARS assay was performed to detect the capacity of the free ligand and its complexes to inhibit lipid peroxidation using ascorbic acid as a standard. All components showed a significant inhibitory effect against oxidative stress at p < 0.01. All the complexes significantly diminished the TBARS level compared to their parent ligand (Table 5, Fig. 9), explaining the ability of these compounds to reverse oxidative stress. The ligand, metal complexes and ascorbic acid exerted their radical inhibitory effects in a concentration-dependent manner. Our results showed that chelation with metal ions is effective in the termination of lipid peroxidation. Nickel complexes exhibited the highest inhibition of the TBARS ratio (IC50 = 0.26 mg/mL), while cobalt showed the lowest percentage (IC50 = 0.48 mg/mL) at p < 0.01. The inhibition in the levels of TBARS may reflect the antioxidant capacity of these compounds.

**Table 5:** The effect of ligand and its complexes on antioxidant reducing power at different concentrations (0.05–0. 5 mg/mL).

Concentration	Parameters					
	Ligand	CO	Cu	Zn	Ni	Standard
0.05	0.10 ± 0.01	0.19 ± 0.02	0.25 ± 0.01	0.32 ± 0.01	0.40 ± 0.01	0.72 ± 0.02
0.1	0.13 ± 0.001	0.22 ± 0.001	0.28 ± 0.01	0.36 ± 0.01	0.45 ± 0.01	1.12 ± 0.04
0.2	0.17 ± 0.01	0.25 ± 0.01	0.32 ± 0.01	0.40 ± 0.01	0.49 ± 0.02	1.34 ± 0.04
0.3	0.20 ± 0.01	0.30 ± 0.01	0.37 ± 0.01	0.44 ± 0.02	0.52 ± 0.01	1.52 ± 0.04
0.4	0.23 ± 0.001	0.32 ± 0.01	0.40 ± 0.01	0.49 ± 0.01	0.59 ± 0.01	1.76 ± 0.04
0.5	0.29 ± 0.01	0.37 ± 0.01	0.45 ± 0.01	0.54 ± 0.01	0.79 ± 0.01	1.94 ± 0.05

#### Reducing power

**Figure 10:**
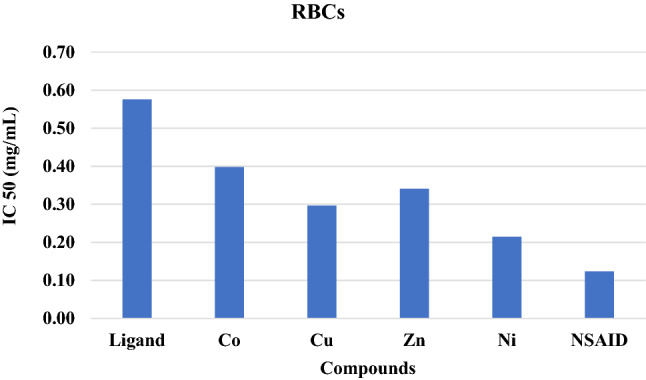
RBC IC50 values for the standard (NSAID), ligand and its metal complexes.

Iron plays an essential role in several biochemical processes, including drug metabolism, cell respiration and oxygen transport. However, iron is also involved in various biochemical oxidation reactions, which are implicated in pathological disorders such as atherosclerosis and neurodegeneration. Therefore, any compound that interacts with iron and stops its oxidative reactions with biological molecules can be used as an antioxidant agent. Compounds that have iron reducing power and act as iron chelating agents can be used for the treatment of ferric-induced diseases such as hemochromatosis, which results in Fe^3+^ accumulation. The Schiff base ligand and its metal complexes could be used as antioxidants to stress the oxidative damage induced by iron^44^. The reducing power reflects the capacity of compounds to donate electrons, modulate the oxidation/reduction reaction of the radicals and reflect their antioxidant activity. In the ferric ion reducing antioxidant power assay, the increase in the absorbance indicates an increase in the reducing capacity of the antioxidant compounds^41^. Generally, the reducing properties depend on the presence of the reductant. The ferric reducing power mechanism responsible for antioxidant properties explained the effect of the compound on the reduction of Fe(III) to Fe(II) to evaluate the antioxidant capacity^52,53^. The Schiff bases had a potent Fe^3+^ reducing activity and electron donor properties for stabilizing and neutralizing free radicals and reactive oxygen species^54^. Figure 10 shows that the synthesized compounds and ascorbic acid changed the ferric yellow color to various shades of blue at 700 nm, depending on the reducing capacity of each compound. The higher absorbance indicates the stronger reducing abilities and antioxidant activity of the samples. The Schiff base ligand exerted a significantly (p < 0.01) lower reducing power than metal complexes, Table 6. Among the complexes, the nickel metal complex showed the highest significant reducing activity (better Fe^2+^-chelator) at p < 0.01. The increase in absorbance of our compounds indicates their ability to reduce Fe^3+^ ions, which may be due to their ability to donate electrons. According to the results, ascorbic acid showed the highest reducing activity when compared to the Schiff base ligand and its complexes. All the components exhibited strong concentration-dependent antioxidant scavenging properties in agreement with those reported in the literature^55^, where Schiff base metal complexes have a stronger reducing capacity than the ligand depending on their concentration.

**Table 6:** The effect of ligand and its complexes on antioxidant RBCs at different concentrations (0.05–0. 5 mg/mL).

Concentration	Parameters					
	Ligand	CO	Cu	Zn	Ni	Standard
0.05	11.76 ± 1.01	19.52 ± 0.64	27.48 ± 0.52	24.43 ± 1.04	35.06 ± 0.15	44.41 ± 0.76
0.1	19.82 ± 0.75	26.97 ± 1.21	33.66 ± 0.75	29.30 ± 0.72	41.66 ± 1.0	49.44 ± 0.83
0.2	26.93 ± 0.44	34.84 ± 0.90	42.28 ± 0.73	38.57 ± 0.86	48.65 ± 0.86	55.96 ± 1.25
0.3	32.67 ± 0.80	45.35 ± 0.67	52.55 ± 0.77	49.27 ± 1.11	56.64 ± 0.84	62.46 ± 0.96
0.4	38.48 ± 1.16	50.58 ± 0.81	59.27 ± 0.96	55.88 ± 1.31	66.57 ± 0.61	69.69 ± 0.62
0.5	43.42 ± 0.68	55.72 ± 0.86	65.43 ± 0.71	61.17 ± 1.35	73.56 ± 0.60	83.29 ± 0.53

### AChE activity

**Figure 11:**
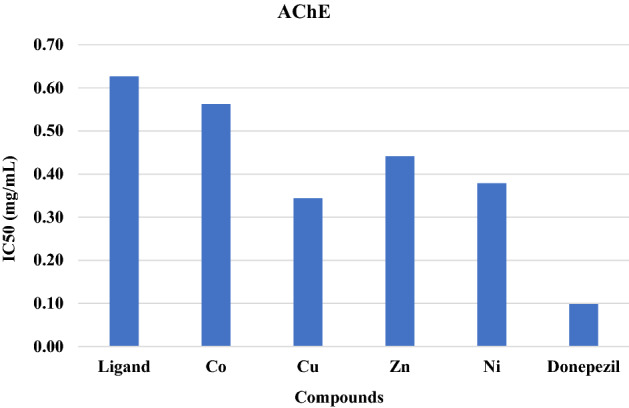
RBC IC50 values for the standard (donepzil), ligand and its metal complexes.

Acetylcholine esterase (AChE) is mainly found in the central nervous system (CNS) and hydrolyzes the neurotransmitter acetylcholine (ACh) to choline. The hyperactivation of AChE and ACh deficiency is associated with cholinergic neuron dysfunction and abnormal neurotransmission. Therefore, AChE hyperactivity plays a pathogenic role in the induction of many neurodegenerative disorders such as Alzheimer’s disease (AD). Pharmacological research for drug screening to resist AD pathogenesis has focused on AChE suppression to improve neurotransmission and cholinergic deficits. Some Schiff bases can inhibit AChE, improving neurotransmission. Acetylcholine esterase inhibitors boost memory and cognitive functions and have been considered a strategy for the therapy of dementia and AD^56^. The free ligand and its complexes showed a significant (p < 0.01) inhibitory effect against AChE (Table 7, Fig. 11). All the tested compounds inhibited the enzyme in a concentration-dependent manner. The copper complex exhibited the strongest inhibitory effect with IC50 = 0.34 mg/mL, while cobalt showed a lower AChE inhibitory effect with IC50 = 0.56 mg/mL. These results are in concordance with the literature^57^, where the Schiff base ligand and its complexes (Fe, Ru, Co and Pd) have inhibitory effects against AChE activity.

**Table 7:** The effect of ligand and its complexes cobalt (Co), copper (Cu), zinc (Zn) and nickel (Ni) on antioxidant AChE at different concentrations (0.05–0.5 mg/mL).

Concentration	Parameters					
	Ligand	CO	Cu	Zn	Ni	Standard
0.05	17.70 ± 1.04	20.27 ± 1.15	28.27 ± 0.80	22.47 ± 0.80	26.20 ± 0.85	47.60 ± 0.61
0.1	21.83 ± 1.5	25.50 ± 1.15	35.23 ± 0.81	27.93 ± 1.38	32.73 ± 1.46	58.1 ± 0.95
0.2	27.27 ± 1.16	31.30 ± 1.01	42.17 ± 1.79	36.63 ± 1.26	39.53 ± 0.85	67.07 ± 1.48
0.3	32.90 ± 1.61	36.60 ± 0.75	48.43 ± 0.80	42.53 ± 0.85	45.60 ± 0.89	78.83 ± 1.14
0.4	38.57 ± 1.0	41.47 ± 0.76	54.27 ± 1.07	47.33 ± 1.1	51.37 ± 1.36	87.50 ± 0.96
0.5	41.97 ± 1.08	45.43 ± 0.65	58.77 ± 0.81	52.13 ± 0.85	56. 3 ± 0.75	93.67 ± 1.37

### Membrane stabilization activities

In several pathological disorders, such as thalassemia, sickle cell anemia and malaria, RBC membranes are hemolyzed, releasing their hemoglobin^58,59^. Erythrocyte is very sensitive to oxidative stress and hemolysis due to its high concentration of oxygen and high polyunsaturated content. Therefore, antioxidant supplementation might strengthen the radical defense system of RBCs^60^. In this result, the Schiff base ligand and its metal complexes significantly (p < 0.01) suppressed the erythrocyte membrane lysis induced by hypotonic solution, offering strong protection against RBC hemolysis and cell damage induced by inflammatory agents. The nickel complex showed the highest antihemolytic activity toward RBCs (IC50 = 0.21 mg/mL). The standard NSAID showed a significantly higher antihemolytic activity (IC50 = 0.12 mg/mL) than the Schiff base compounds. All the compounds exhibited concentration-dependent effects. Our synthetic compounds can improve the integrity of the cells and stability of their membranes. The membrane stabilizing activity of these compounds may be related to their antioxidant capacity to protect against cytotoxicity.

### Total antioxidant capacity

**Figure 12:**
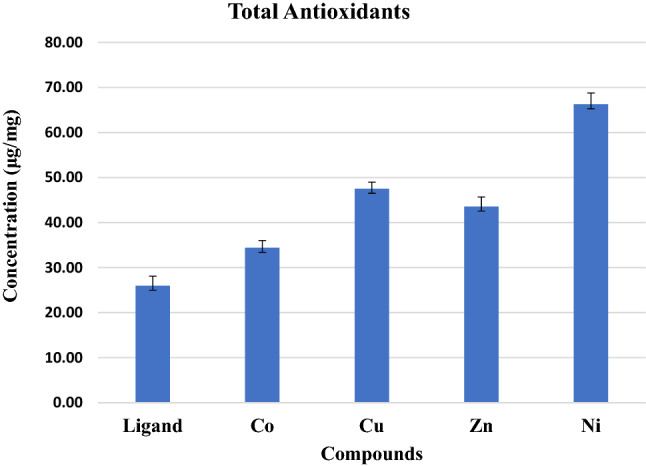
Total antioxidant concentration (μg/mg) for the ligand and its metal complexes.

The antioxidant substances are capable of counteracting the damage caused by oxidative stress due to free radical propagation. Natural and synthetic antioxidants are used to protect against oxidant molecules and delay their deterioration. Additionally, antioxidants repair the risk of several diseases, including cancer, atherosclerosis, diabetes, eye disorders, autoimmune diseases, neurodegenerative disorders, and aging diseases^53^. In the current study, the total antioxidant activity of the Schiff base ligand was significantly (p < 0.01) lower than that of all the complexes (Fig. 12), presenting protection against oxidative stress-induced tissue damage. Among these complexes, the Ni complex exhibited a stronger total antioxidant capacity (66.28 μg/mg ± 2.51) than the other complexes (Zn, Cu and Co) at p < 0.01. These results are in line with those reported in the literature^61^, which demonstrated that the total antioxidant activity of Schiff base ligands was lower than that of Cu complexes through the phosphor molybdenum experiment and that the total antioxidant ability of Schiff base ligands and their metal complexes (Ni, Co, Cu, and Zn) was dose-dependent in the molybdenum assay at different concentrations. The existence of a Schiff base and -OH groups also has an impact on the DPPH radical scavenging efficiency. Antioxidant mensuration from the attended compounds explained that the OH functional groups as well the existence of electron donation significantly impacted the radical scavenging efficiency from phenolic Schiff bases. The higher antioxidant activity of the complexes is due to the acquisition of additional superoxide dismutation centers, which causes an increase in the molecule’s ability to stabilize unpaired electrons and therefore to scavenge free radicals. Cu^+2^ and Zn^+2^, Co^+2^ ions coordinated to the keto enol functionality of the prepared ligand alter the antioxidant activity of the prepared ligand. Spectroscopic investigations of the complexes point towards metal coordination at the keto enol moiety of the prepared ligand. Thus, the hydroxy groups can participate in free radical scavenging activity. Moreover, vitamin C has the highest total antioxidant capacity compared to Schiff base ligands and their complexes^61^.

In comparison with other related analogs, the antimicrobial activities of transition metal complexes with *N,N*’-bis (1-naphthaldimine)-*p*-oxydianiline (H_2_L) were tested against the same examined pathogenic microorganisms^10^. H_2_L proved to be inactive, while zinc, copper, and nickel complexes exhibited moderate antibacterial activity compared to the standard antibiotic. Furthermore, the zinc complex specifically displayed high activity toward *P*. *aeruginosa*, *S*. *aureus* and *S*. *faecalis*, while the copper complex was only active against *E*. *coli* and *S*. *aureus*. The cobalt complex has no detectable efficacy against all investigated microbes. The variation in activity of different metal (II) complexes against organisms depends either on the impermeability of the cells or differences in the ribosome. The octahedral nickel complex showed obvious inhibition of *C*. *albicans*, whereas other complexes were inactive. The antifungal activity may be dependent on both the geometry and the bulk of the complex. On the other hand, the antioxidant activity studies indicated that free radical scavenging of the ligand and its complexes with the DPPH radical revealed the weak antioxidant power of the ligand. However, metal complexation significantly enhanced scavenging, and the activity was concentration dependent. The scavenging potential may be related to the reducing action of the free radical to convert it into a nonreactive species. The NO scavenging effects clearly indicated that complexes were more significant than the ligand, and the suppression ratio increased with increasing concentration. The zinc complex was the most effective among all the complexes and showed slightly less antioxidant activity than the standard ascorbic acid. The reducing power effect indicated that the zinc complex exhibited the highest activity, while cobalt metal showed the lowest reducing ability, and the power increased with increasing concentration. The reducing ability of a compound generally depends on the development of reductones, which display antioxidative potential by terminating the free radical chain reaction. The inhibitory effects against AChE indicated that all compounds inhibited the enzyme in a concentration-dependent manner at p < 0.005. The nickel complex exhibits the strongest inhibition compared to other compounds. All complexes exhibited high antihemolytic activity toward human erythrocytes, and the fraction of hemolysis inhibition was concentration dependent. In comparison to other complexes, the copper complex exhibited the highest activity. In view of these findings, the antioxidant activity of the complexes depends on both the oxidation state of the metal and the ligand type. The redox properties of such complexes depend on several factors, such as the degree of unsaturation and the size of the chelate ring.

Antimicrobial studies of transition metal complexes with *N,N*’-bis (1-naphthaldimine)-*p*-sulphonyldianiline (H_2_L)^7^ proved that the ligand has no efficacy against investigated microbes. The tetrahedral Ni(II) complex exhibited moderate activity towards all the studied microbes, while other complexes showed a negative effect. The increased activity of nickel chelate can be explained on the basis of the overtone concept and chelation theory, in which nickel chelate deactivates various cellular enzymes that play a vital role in various metabolic pathways of these microorganisms. However, the variation in activity between the tetrahedral nickel complex and other inactive metal(II) complexes may be related to the nature and structure, where only one nickel center is present, and this may increase the lipophilic nature and allow it to react easily with the bacterial cell wall. The complexes exhibited potent antioxidant and anti-inflammatory activities in the order [Cu_2_L_2_]0.4H_2_O > [Zn_2_L_2_] > [Ni (HL)_2_] > [Co_2_L_2_] > H_2_L. The DPPH scavenging activity indicated that metal chelation significantly enhanced the antioxidant activity. The copper complex exhibited high scavenging activity, whereas cobalt showed low activity. The antioxidant activity of the complexes was low compared to that of ascorbic acid, except in the case of the copper complex, which exhibited activity similar to that of vitamin C. The complexes showed powerful inhibitory action against oxidative stress in liver tissue. All the complexes significantly reduced the TBARS level compared to their parent ligand. Notably, the copper complex exhibited the highest TBARS activity, whereas the TBARS activity of the cobalt complex was low. TBARS inhibition in the liver tissue explained the reduction in oxidative stress, which may be related to the antioxidant effect of these compounds. The increase in TBARS levels is associated with a reduction in antioxidant enzymes such as superoxide dismutase (SOD), glutathione peroxidase (GPX), catalase (CAT) and glutathione reductase (GR). SOD is considered the first line of defense against free radicals through catalyzing the dismutation of superoxide anion radical (O_2_^-^) into molecular oxygen. Numerous types of SOD are found in mammalian cells and have metals in their active sites, such as Cu/Zn, Ni, Fe or Mn. With respect to normal cells, cancer cells produce a large amount of O_2_^⋅−^ and the activity of SOD in cancer cells is lower than that in normal cells. The studies of the complexes to activate hepatocyte SOD suggested that these compounds acted as SOD activators and that the complexes manifested SOD activity better than the ligand. The SOD activity of H_2_L and the complexes was correlated with the concentration, and the results showed that the inhibition of the superoxide anion ratio increased with increasing sample concentration. Both zinc and copper complexes exhibited better superoxide anion scavengers than nickel and cobalt complexes. The inhibitory effect of the complexes on hepatocyte lipid peroxidation stimulated by superoxide anion is useful to develop a new antioxidant generation for liver diseases.

## Conclusions

We report a modified ecofriendly synthesis and a proposed reaction mechanism of the ligand 4,4’-methanedianiline mediated by natural acidic kaolinite clay. Condensation of the diamine with two equivalents of the commercial 2-hydroxy-1-naphthaldehyde produced a (1:1) inseparable mixed Schiff base ligand that appeared as an Octopus-like morphology as judged by the SEM image. The obtained mixed ligand imine was reacted with four transition metal salts, namely, Co(OAc)_2_⋅H_2_O, NiCl_2_⋅6H_2_O, Cu(OAc)_2_. H_2_O and Zn(OAc)_2_⋅2H_2_O furnished their corresponding complexes in high yield and purity. The structures of the ligand and its metal complexes were fully characterized by spectroscopic and spectrometric measurements. All complexes exhibited high thermal stability up to 700 °C, leaving, in most cases, approximately 40% of their mass as residues. Antimicrobial screening results of the ligand and its metal(II) complexes indicated that zinc and nickel complexes exhibited moderate activities towards all studied microbes, while the ligand and the cobalt complex had no efficacy. Antioxidant screening was concentration dependent, and their activities were in the order Ni(II) > Zn(II) > Cu(II) > Co(II) complexes. The NO inhibitory effect of the ligand and its metal complexes was concentration dependent, and the nickel complex exhibited the highest activity, whereas the cobalt complex showed the lowest inhibition. All components showed a significant lipid peroxidation inhibitory effect against oxidative stress at p < 0.01. All the complexes significantly diminished the TBARS level, and the nickel complex exhibited the highest inhibition (IC50 = 0.26 mg/mL), while the cobalt complex showed the lowest percentage (IC50 = 0.48 mg/mL) at p < 0.01, and this inhibition level reflects the antioxidant capacity of these complexes. Evaluation of the ligand and its complexes as antioxidants for stressing the oxidative damage induced by iron indicated that the ligand exerted a significantly lower reducing power than the complexes and that the activities were concentration dependent. Among the complexes, the nickel complex showed the highest reducing activity. The free ligand and its complexes showed an interesting inhibitory effect against acetylcholine esterase in a concentration-dependent manner. The copper complex exhibited the highest activity, whereas the cobalt complex showed the lowest inhibition. Screening of membrane stabilization activities of the ligand and its complexes clearly indicated that most compounds can improve the integrity of the cells and stability of their membrane, and this result may be related to their antioxidant capacity to protect against cytotoxicity. In general, the antioxidant activity of the ligand was lower than that of its complexes. The nickel complex exhibited a stronger total antioxidant capacity than the other complexes.

## Supplementary Information

Supplementary Figures.

## Data Availability

All data generated or analyzed during this study are included in this published article and supplementary materials.
