# Author Correction: Green synthesis of nanosized *N,N*'-bis(1-naphthylidene)-4,4'- diaminodiphenylmethane and its metal (II) complexes and evaluation of their biological activity

**DOI:** 10.1038/s41598-023-29769-5

**Published:** 2023-02-14

**Authors:** Hammed H. A. M. Hassan, Hend M. Hussein, Amel F. Elhusseiny

**Affiliations:** 1 Department of Chemistry, Faculty of Science, Alexandria University, Moharram Beck, P.O. Box 2, Alexandria, 21568 Egypt; 2 Pharmacology and Therapeutics Department, Faculty of Pharmacy, Pharos University, Canal El Mahmoudia Street, Alexandria, 21311 Egypt

Correction to: *Scientific Reports* 10.1038/s41598-022-25650-z, published online 07 December 2022

The original version of this Article contained an error in the spelling of the author Hend M. Hussein, which was incorrectly given as Hend Hussien.

Additionally, the original version of this Article contained an error in Affiliation 2, which was incorrectly given as ‘Department of Pharmacology and Toxicology, Faculty of Pharmacy, Pharos University, Canal El Mahmoudia Street, Alexandria, 21311, Egypt’. The correct affiliation is listed below:

Pharmacology and Therapeutics Department, Faculty of Pharmacy, Pharos University, Canal El Mahmoudia Street, Alexandria 21311, Egypt.

The original Article and accompanying Supplementary Information file have been corrected.

