# Supplementary material for: Green synthesis of nanosized N,N'-bis(1-naphthylidene)-4,4'-diaminodiphenylmethane and its metal (II) complexes and evaluation of their biological activity
Source: Sci Rep. 2022 Dec 7;12:21142. doi: 10.1038/s41598-022-25650-z (PMC9729294; doi:10.1038/s41598-022-25650-z)

Green synthesis of nanosized N,N'-bis(1-naphthylidene)-4,4'- diaminodiphenylmethane and its metal (II) complexes and evaluation of their biological activity

Hammed H. A. M Hassan*[a], Hend M. Hussein[b], Amel F. Elhusseiny[a]

[a]*Department of Chemistry, Faculty of Science, Alexandria University, P.O. Box 2-Moharram Beck, Alexandria-21568, Egypt.* [b]*Pharmacology and Therapeutics Department, Faculty of Pharmacy, Pharos University, Canal El Mahmoudia Street, Alexandria 21311, Egypt.*

....

Supplementary materials

N,N'-Bis(1-naphthylidene)4,4'-diaminodiphenylmethane **3**

IR

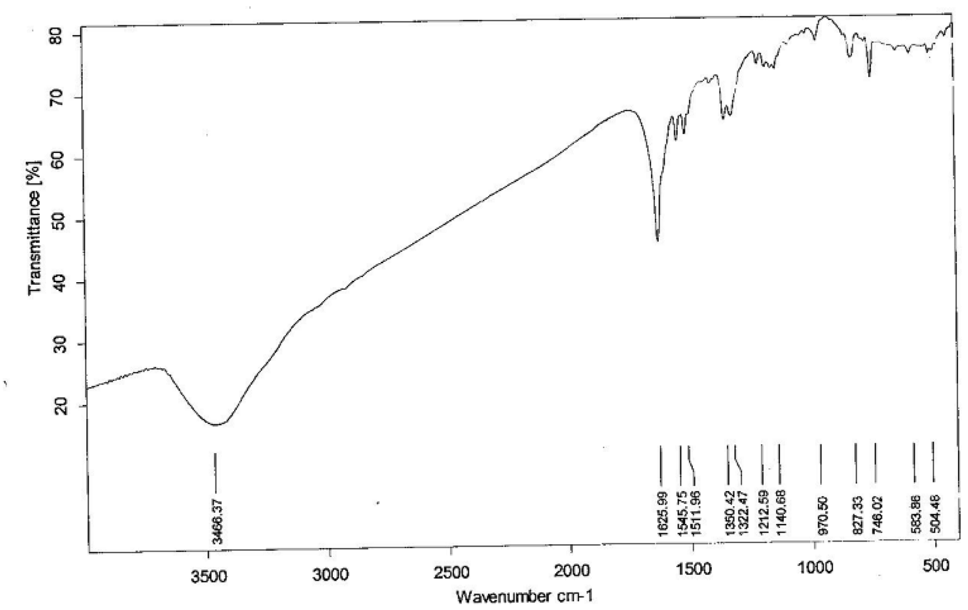

MS

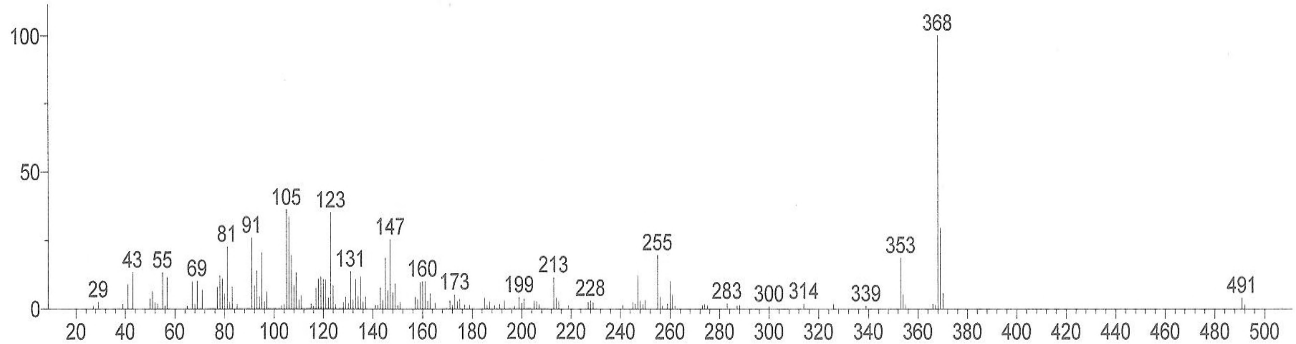

^{1}HNMR

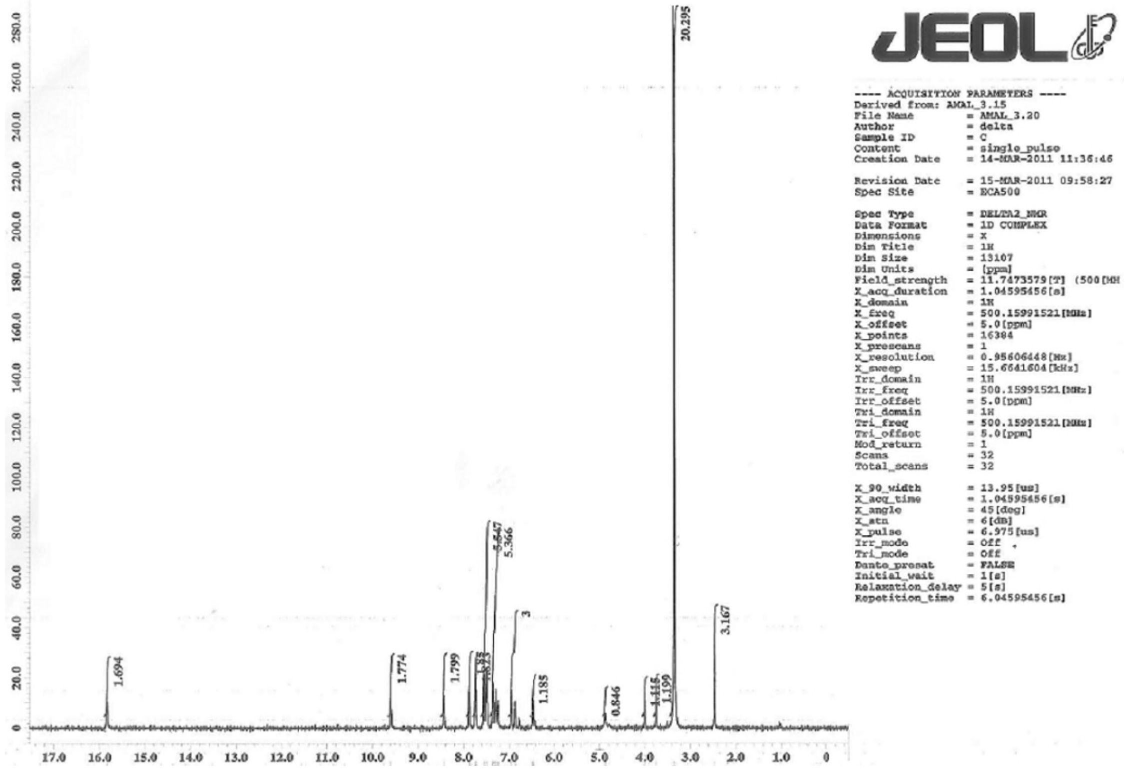

^{13}C NMR

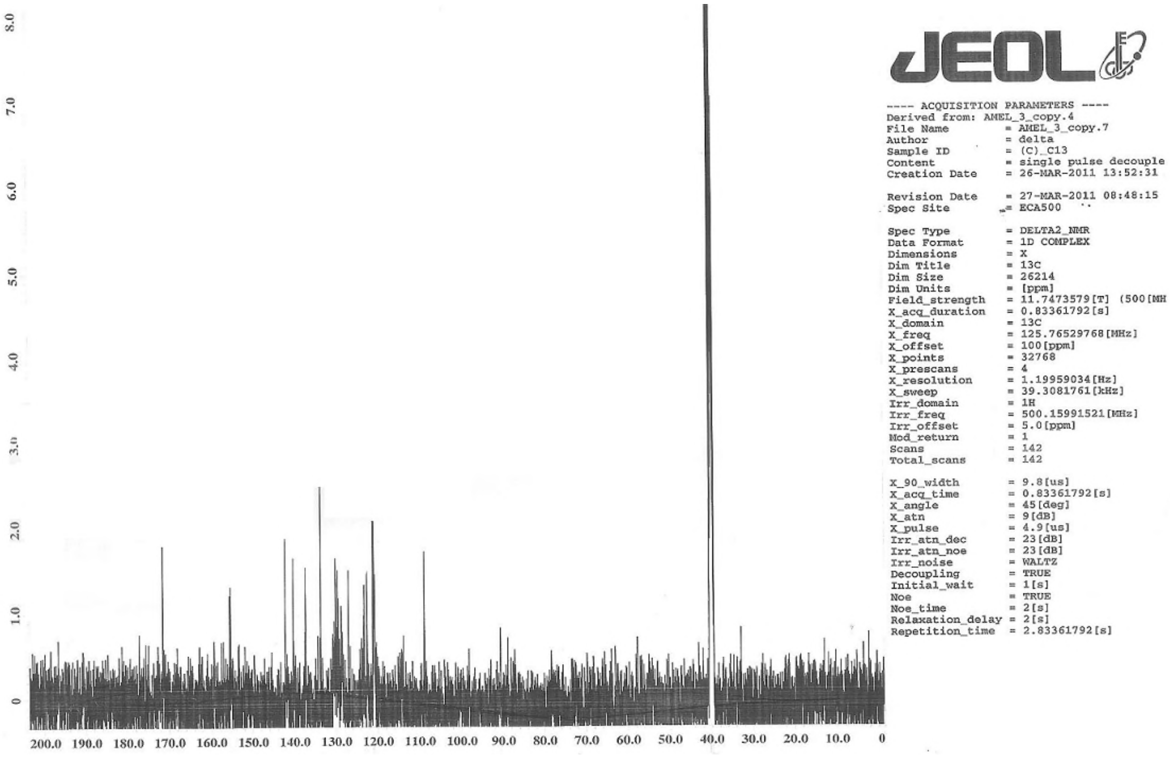

....

Zinc complex **5**

IR

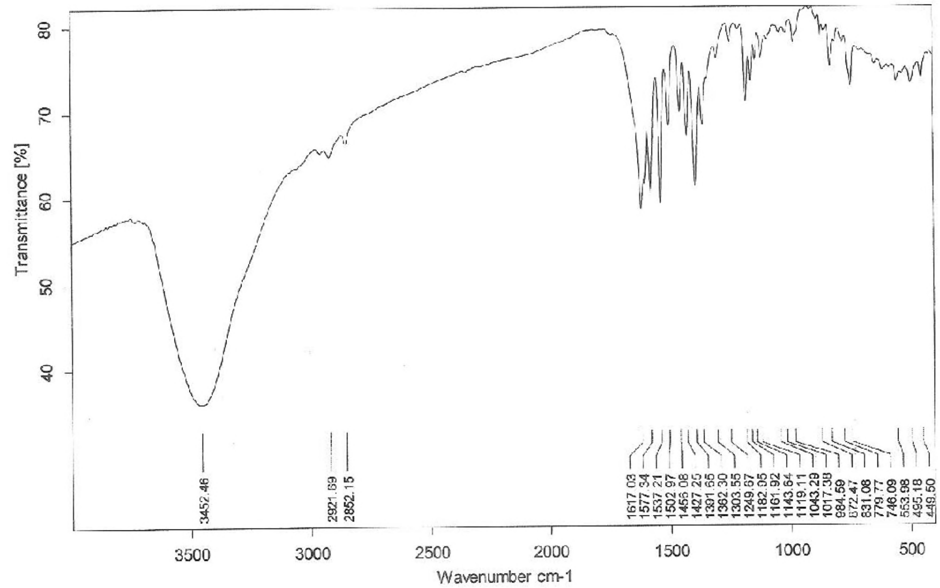

1H NMR

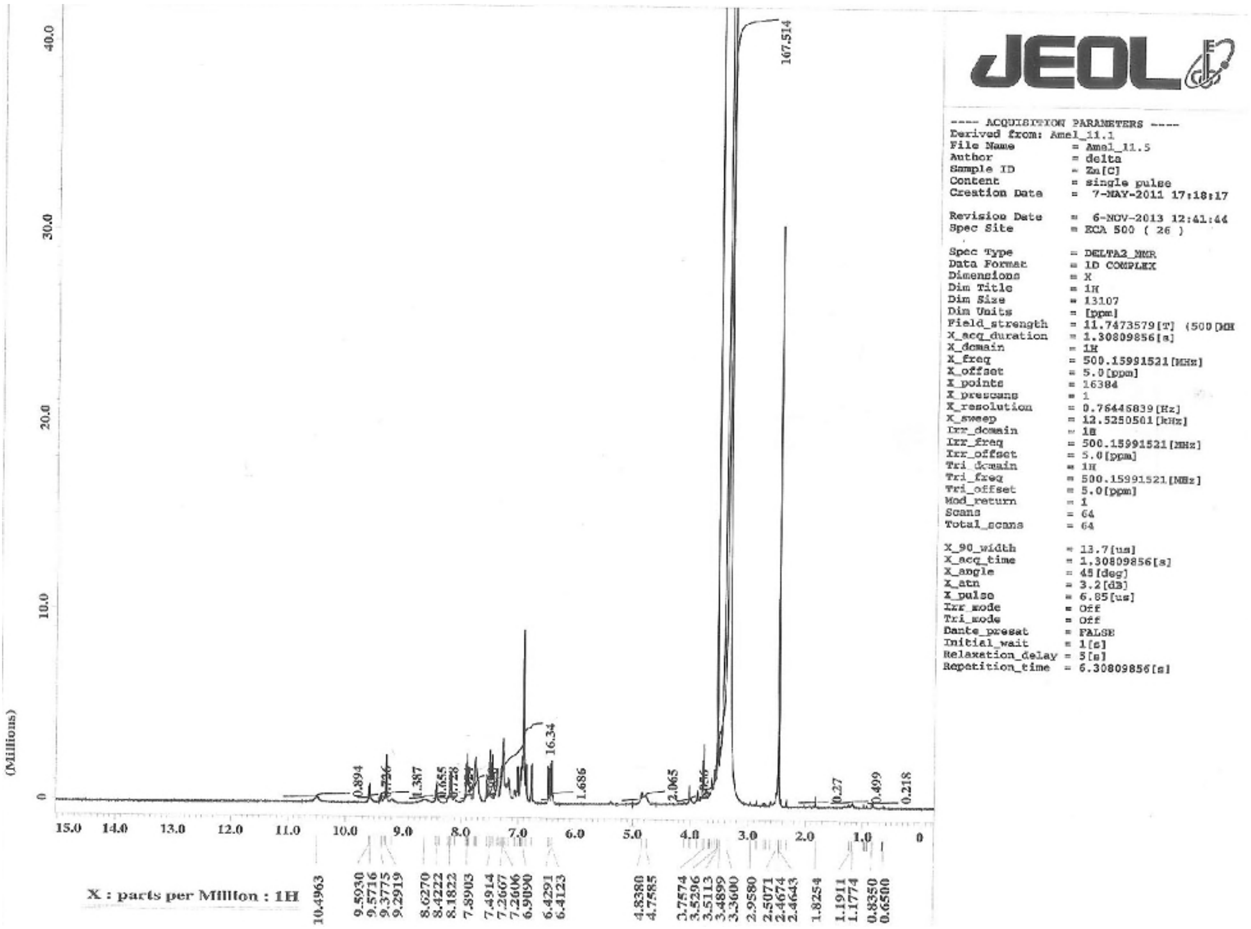

Cobalt complex **6**

IR

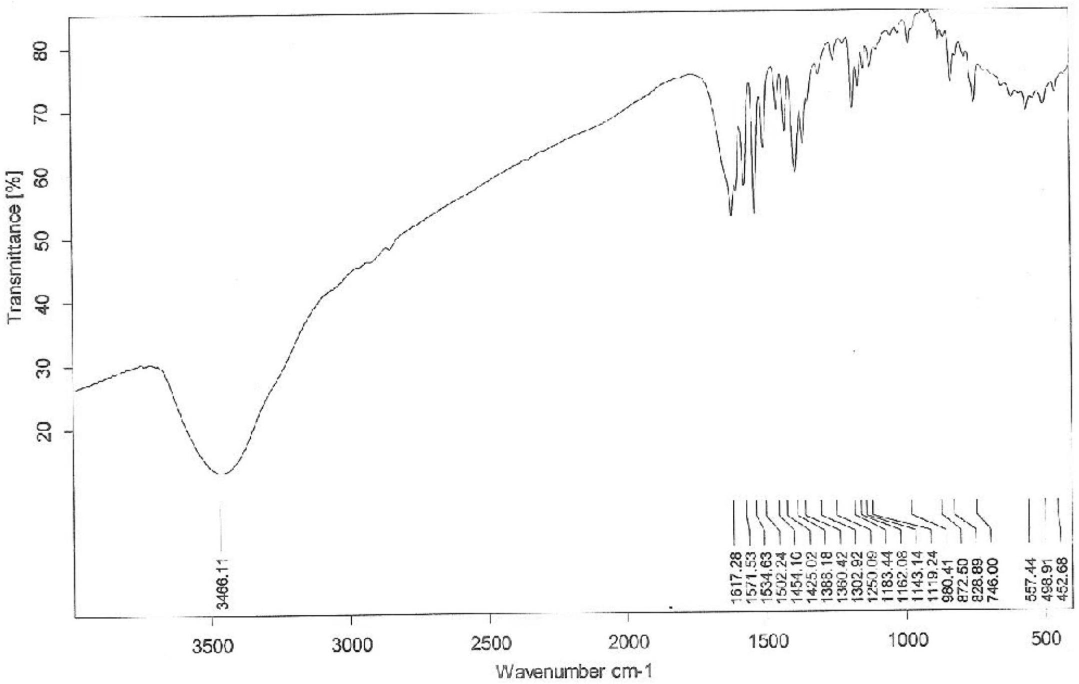

MS

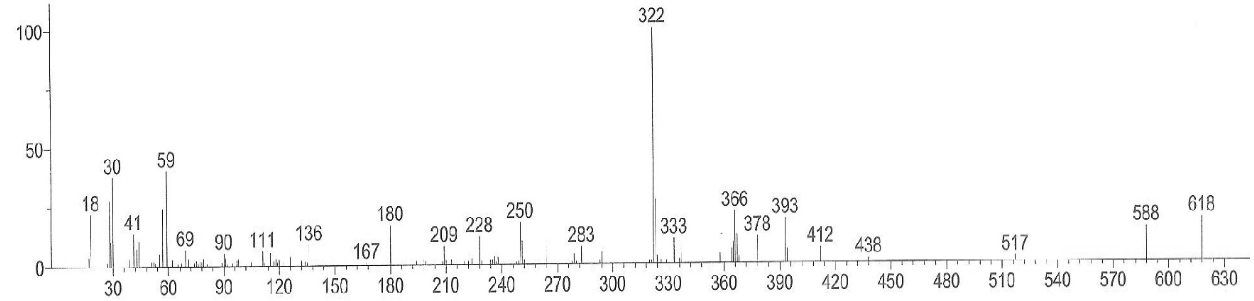

Copper complex **7**

IR

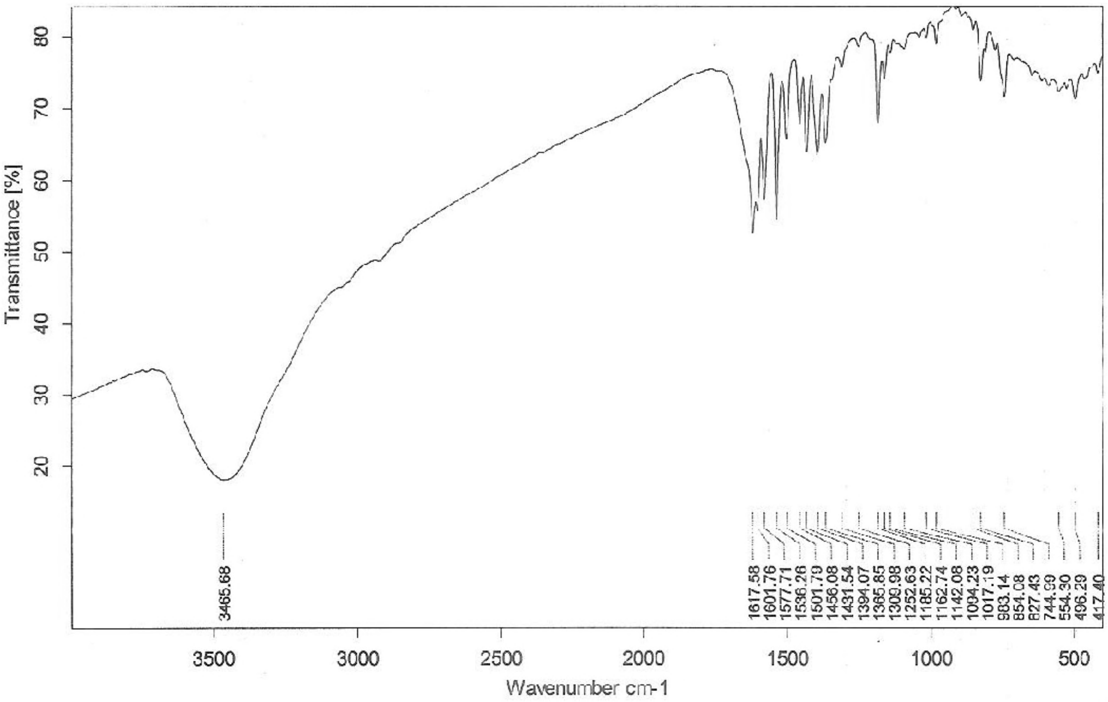

MS

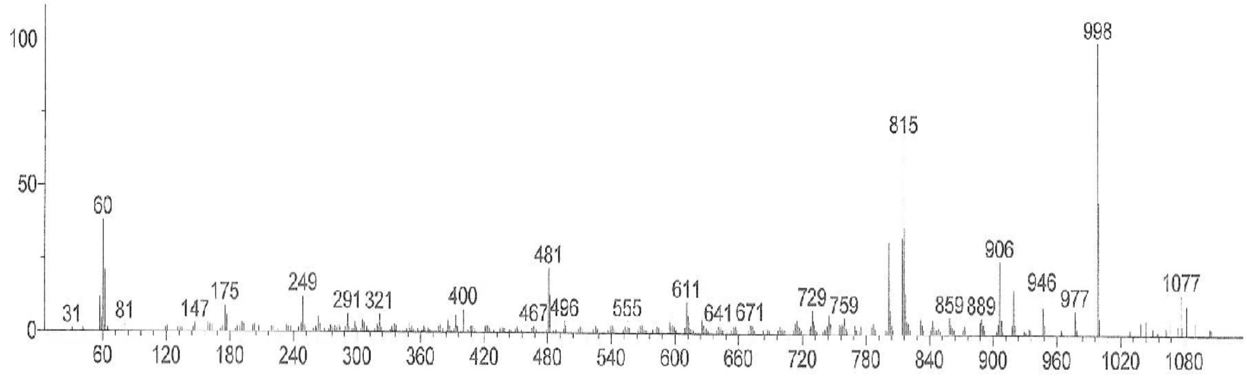

Nickel complex **8**

IR

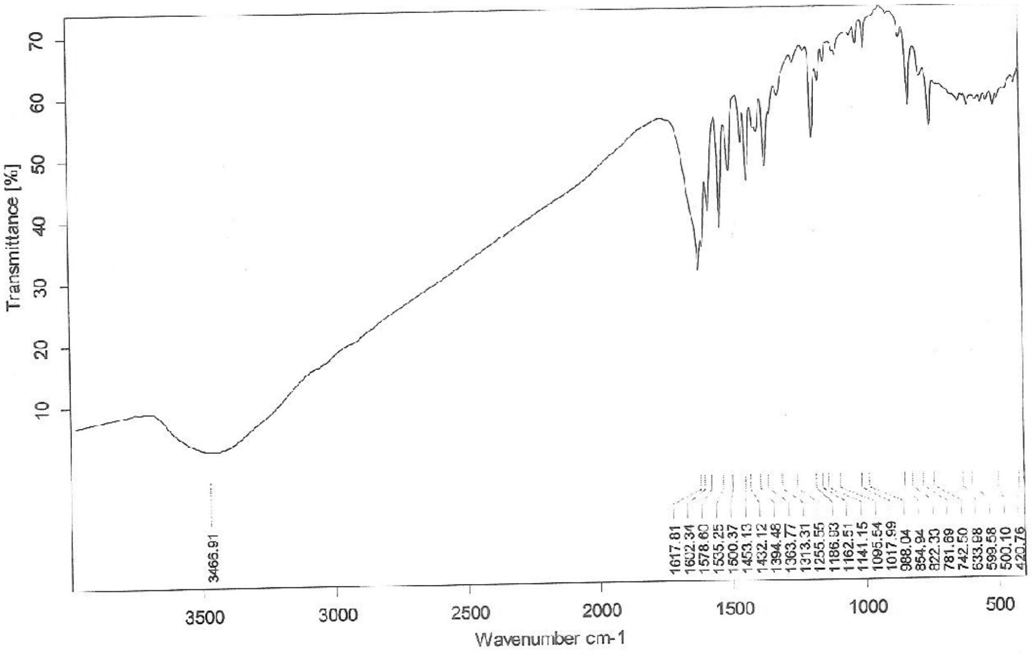

MS

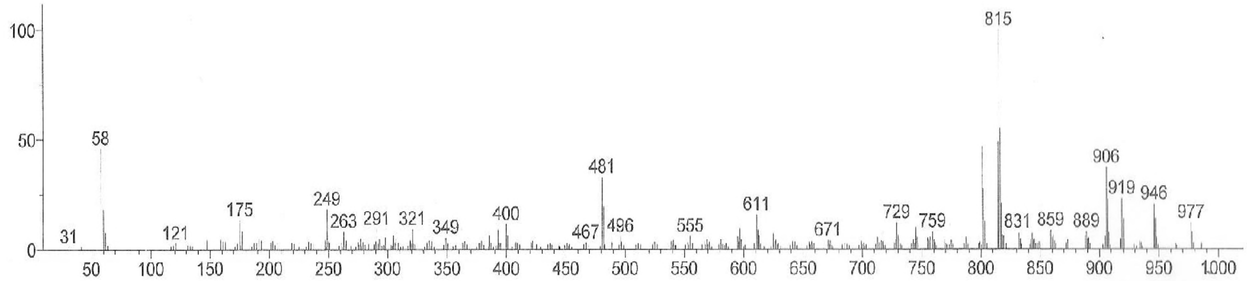

Supplement: Supplementary file 1 — Supplementary Figures. [file 41598_2022_25650_MOESM1_ESM.pdf]
